# Gut microbiota and western dietary patterns associated with behavioral problems in children and adolescents: a cross-sectional study

**DOI:** 10.1186/s12937-026-01335-5

**Published:** 2026-05-25

**Authors:** A. Larroya, S. Romera-Giner, V. Tolosa-Enguís, S. M. Rodríguez-Ruano, S. Andrés-García, I. Soro-Conde, P. Codoñer, Y. Sanz

**Affiliations:** 1 Microbiome Innovation in Nutrition & Health, Institute of Agrochemistry and Food Technology, Spanish National Research Council (IATA-CSIC), Avda. Agustin Escardino, 7, Valencia, Paterna 46980 Spain; 2 Neurological Rehabilitation Center, Limbic, Valencia, 46015 Spain; 3 Deparment of Pediatrics, Dr. Peset University Hospital, Avd. De Gaspar Aguilar, 80, Valencia, 46017 Spain; 4 Deparment of Pediatrics, Obstetrics and Gynecology, University of Valencia, Valencia, Spain

**Keywords:** Microbiota, Gut-brain axis, Behavior, Western diet, Fibers, Short-chain fatty acids

## Abstract

**Background:**

Childhood and adolescence are crucial periods for brain development, during which multiple environmental factors, including gut microbiota and dietary habits, play important roles. However, the combined impact of those factors on neurodevelopment and mental disease risk remains largely unexplored. Here, we aimed to investigate the relationships between gut microbiota and diet and their role in classifying behavioral problems that may precede mental disorders in children and adolescents.

**Methods:**

We performed a cross-sectional study, including data from 335 subjects, including 202 children (5–10 years) and 133 adolescents (11–17 years). Gut microbiota was analysed in stools by shotgun metagenomics. Dietary habits, lifestyle factors and emotional and behavioral difficulties were screened using validated questionnaires. Penalized Logistic Regression models were trained to classify individuals into Healthy and Behavioral Problem groups based on microbial diversity, differential abundance of bacterial species, dietary patterns, and food and nutrient intakes. Mediation analyses were applied to assess whether gut microbiota mediates the effect of diet on behavioral problems.

**Results:**

A Western diet characterized by poor adherence to dietary recommendations was consistently associated with behavioral problems in all age groups. Individuals with behavioral problems exhibited distinct gut microbiota profiles characterized by lower levels of short-chain fatty acid-producing bacteria (particularly butyrate-producing species) and higher levels of potential pathogens (e.g., *Campylobacter coli* and *Lautropia mirabilis*), linked to poor dietary choices. Furthermore, we evidenced the mediation role of the gut microbiota in the association between dietary patterns and food groups and behavioral problems. In adolescents, *L. mirabilis* was identified as a mediator of the relationship between a Western diet and behavioral problems, while *Anaerostipes rhamnosivorans* mediated the relationship between fish consumption and behavioral problems. Gut microbiota data enhanced the classification accuracy of logistic regression models for identifying individuals with behavioral problems over models based solely on dietary data.

**Conclusion:**

Integrating dietary habits and gut microbiota data enables more accurate stratification of children and adolescents at risk for behavioral problems. Our findings may help to refine dietary interventions targeting the gut microbiota to improve mental health outcomes in these vulnerable populations.

**Supplementary Information:**

The online version contains supplementary material available at 10.1186/s12937-026-01335-5.

## Introduction

The 'gut-brain axis' describes a complex bidirectional communication network linking the gut microbiota and the brain, involving neural, neuroendocrine, metabolic, and neuroimmune pathways [1, 2]. Studies in germ-free mice have provided compelling evidence for the profound impact of the microbiota on these pathways, influencing various aspects of neurodevelopment, including behavior, social abilities, and emotions [3–5].

Gut bacteria also interact with the diet, playing a crucial role in producing neurotransmitters from amino acids (serotonin, ɣ-aminobutyric acid [GABA], etc.) and short-chain fatty acids (SCFAs) from fiber fermentation, which are involved in the modulation of neurophysiology, behavior, and immunity [1, 6]. These observations collectively suggest that the gut microbiota, per se and through dietary interactions, acts as a crucial biological mediator, significantly influencing individual variations in cognitive and behavioral functions [7].

While many clinical studies have explored the influence of the gut microbiota on neurodevelopmental and behavioral disorders in infants and early childhood [8–12], research specifically examining its impact on these disorders during later childhood and adolescence remains understudied. Furthermore, most observational studies linking the gut microbiota to mental health have primarily focused on specific disorders, such as autism spectrum disorder (ASD) and attention-deficit/hyperactivity disorder (ADHD) [13–17]. Fewer studies have investigated the association between the gut microbiota and broader emotional or behavioral difficulties, which may serve as precursors or co-occurring features of various neurodevelopmental and psychiatric disorders [18–20]. While one study found no significant associations between microbiota and behavioral issues in children, others observed links between social anxiety and decreased *Faecalibacterium* abundance in adolescents, and between externalizing behavior and increased *Prevotella* clusters, in both children and adolescents [19, 20].

It is well-established that dietary factors influence both the gut microbiota composition and brain function [21]. For example, certain dietary fibers increase SCFA production, fostering a diverse gut microbiota with anti-inflammatory and immunomodulatory characteristics [22, 23]. Furthermore, dietary interventions have the potential to regulate levels of the inhibitory neurotransmitter GABA by modifying the gut microbiota, which may exert beneficial effects on mental well-being [24]. Cross-sectional studies have demonstrated links between unhealthy diets, such as the Western diet, and poorer mental health in children and adolescents [25]. However, only one study has simultaneously examined the interplay between dietary factors, gut microbiota, and mental health in a pediatric population aged 2–7 years, albeit specifically ASD symptoms [26]. Accordingly, a comprehensive understanding of how dietary components and patterns impact behavioral problems, particularly through gut-mediated mechanisms, is still lacking in childhood and adolescence.

Thus, the primary aim of this cross-sectional study was to characterize dietary habits and diet quality in children and adolescents and identify potential diet-related differences that might be linked to variations in gut microbiota composition and behavioral problems. To this end, we investigated the associations between gut microbiota, lifestyle factors (particularly diet), and behavioral outcomes, and we assessed whether the gut microbiota mediates the relationship between dietary factors and behavioral problems. We employed advanced shotgun metagenomic analysis to provide a high-resolution characterization of the gut microbiota. Additionally, we assessed the intake of individual nutrients, foods, diet quality indices, and dietary patterns and their relationships with both the gut microbiota and behavioral outcomes using sophisticated statistical methods. Our ultimate goal was to generate hypotheses to be tested in future longitudinal and intervention studies, which may ultimately inform the development of more precise dietary recommendations for young people to improve mental well-being.

## Methods

### Study design and participants

**Fig. 1:**
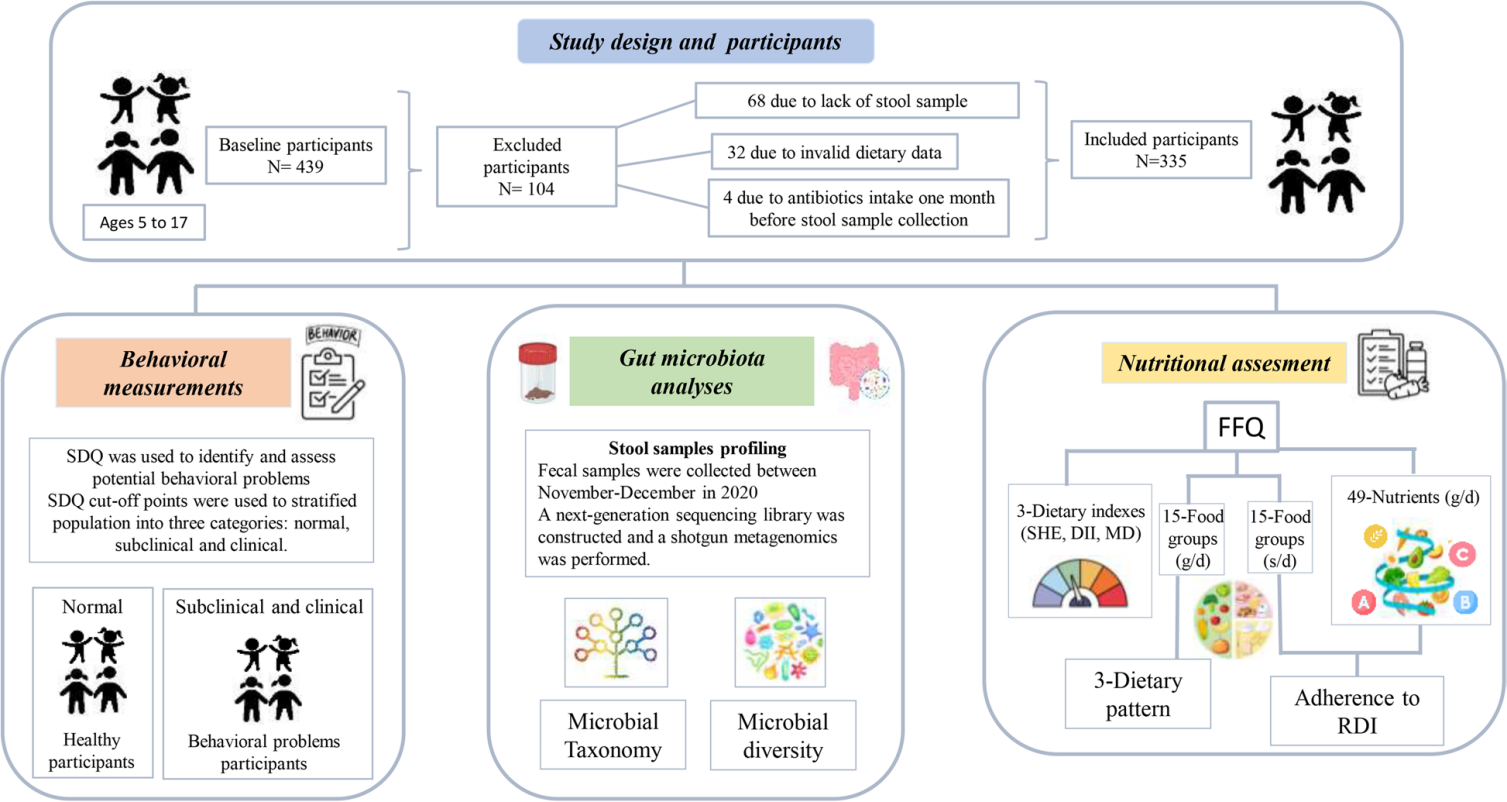
Flow chart of study design, number of participants and data collection. Abbreviations: SDQ: The Strengths and Difficulties Questionnaire; RDI: Recommended daily intake; SHEI: Spanish healthy eating index; DII: Dietary inflammatory index; MD: Mediterranean diet

This cross-sectional study included 335 Spanish participants from a larger population study conducted in 15 schools in Valencia, Spain, encompassing ages 5 to 17. Recruitment occurred between November and December 2020 during the SARS-CoV-2 pandemic, although none of the participants had been infected with the virus at that time, according to specific antigen testing. We excluded participants with chronic inflammatory bowel disease or other chronic conditions affecting the gut. Study design and assessments are depicted in Fig. 1.

### Clinical and lifestyle measures

Parental reports provided data on sociodemographic factors (age, sex, parental education, marital status), pregnancy history (mode of delivery and breastfeeding), bowel habits (assessed using the Bristol Stool Scale), medical history (including respiratory, allergy, gastrointestinal, sleep and neurological disorders), antibiotic or other medication use (categorized by the Anatomical, Therapeutic, Chemical Classification System) and perceived stress levels using the children’s daily stress inventory [27].

Anthropometric measures, including body mass index for-age z-score (z-BMI) and waist circumference (WC), were evaluated using international cut-offs based on z-score curves established by the World Health Organization (WHO) [28]. Physical activity was evaluated through the Physical Activity Level Questionnaire (APALQ) [29] while the Healthy Lifestyle Score (HLS) was analyzed by daily time spent in team sports, outdoor activities, and screen activities [30].

### Behavioral measurements

The Strengths and Difficulties Questionnaire (SDQ) was used as a screening tool to identify and assess potential behavioral or emotional problems. The SDQ evaluates five domains: four are difficulty domains- including emotions, behavior, hyperactivity, and relationship problems with peers- and the last is prosocial behavior. Each domain score ranges from 0 to 10. The first four domains (difficulty domains) are categorized into internalizing (sum of the emotions and peer problem scales) and externalizing (sum of the behavior and hyperactivity), each ranging 0–20. The SDQ total score (sum of the externalizing and internalizing subscales or the four difficulty domains) ranges from 0 to 40. Higher scores indicate more behavioral problems. The population was classified based on SDQ cut-off points established for the Spanish child and adolescent population into three categories: normal (0–15 points), subclinical (16–18 points), and clinical (19–40 points) [31].

### Nutritional assessments

To assess eating habits, parents of participants completed a 137-item semi-quantitative Food Frequency Questionnaire (FFQ). This FFQ was originally developed for the European Prospective Investigation into Cancer and Nutrition (EPIC) [32], and adapted and validated for use with children and adolescents [33]. The consumption of 15 specific food groups consisting of fresh fruit, legumes, vegetables, nuts and olive oil, processed food, pastries and sugars, dairy products, potatoes, whole grain, refined cereals, fish, red meat, white meat, processed meat and sugar beverages was evaluated quantitatively, assessing the amount consumed in servings per day (s/d) and grams per day (g/day), and the intake of 49 micro- and macronutrients was also estimated (g/day). To account for variations in energy intake, we adjusted consumption (g/day) of food groups and nutrients using Willet's residual method.

Participants were classified according to their adherence or not to the daily intake recommendations of the Spanish Society of Community Nutrition (SENC) concerning food groups (s/day), and the dietary guidelines of the Spanish Agency for Food Safety and Nutrition (AESAN) regarding the amount of nutrients consumed (g/day), considering participant age and portion size [34, 35].

Moreover, dietary data were used to calculate the Dietary Inflammatory Index [36] and the Spanish Healthy Eating Index (SHEI) [37], while the adherence to the Mediterranean Diet (MD) was assessed using the validated Kidmed questionnaire [38]. More details are in Additional file 1: Methods S1.

We also conducted a posteriori dietary pattern analysis based on the intake of the 15 assessed food groups. Then we stratified the different patterns identified into tertiles to represent low, moderate or high adherence to each pattern.

### Gut microbiota analyses

#### Stool sampling, DNA extraction, sequencing, and data preprocessing

Stool samples were collected by parents and then stored at −80ºC after delivery at the clinic. Metagenomic DNA samples were isolated from approximately 300 mg of feces using the Magpure Stool DNA LQ Kit (Magen Biotech, Shenzhen, China). A next-generation sequencing library was constructed using the MGIEasy Universal DNA Library Prep Set (MGI Tech., Shenzhen, China) and shotgun metagenomics was performed using the MGI DNBSEQ-G50™ sequencing platform at the MGI Tech Co., Ltd manufacturing facility in Riga (Latvia). This generated paired-end short reads with a maximum of 150 base pairs and a sequencing depth of 20 million reads overage. The resulting reads underwent quality control analysis and subsequent trimming using FastQC (v0.12) [39], MultiQC (v1,13) [40] and Trimmomatic (v0.36) [41]. Reads shorter than 75 bases or with a base quality score below 20 were discarded, as well as the sequences of the corresponding adapters.

To remove human host genome sequences, reads were aligned against the human reference genome (GRCh38) using Bowtie2 (v2.4.5) [42]. The unmapped reads, presumed to be of non-human origin, were converted into FASTQ files and submitted to an additional quality control step, with the aforementioned software and read-discarding criteria, to identify and remove potential artifacts.

Taxonomic assignment was performed using Kraken2 (v2.1.2) [43] with a confidence score threshold of 0.1 and the default Standard Kraken2 database. Subsequently, Bracken (v2.6.2) [44] was used to refine the abundance estimates of bacterial taxa. To facilitate downstream analyses, KrakenTools scripts (v1.2) were used to convert the Bracken output into MetaPhlAn-styled files.

The taxonomic reads were normalized using rarefaction and further filtered for non-human taxa (e.g., eukaryotes and viruses) using the phyloseq R package (v 1.62.2) [45]. Rarefaction depth corresponded to the minimum sample size of the metagenomic dataset, in this case 5240270.

#### Metataxonomic microbiota analysis

Microbial diversity was assessed using alpha diversity (within-sample diversity) metrics such as richness (Observed abundance, Chao1) and evenness (Shannon, Inverse Simpson), and beta diversity (between-sample dissimilarity) metrics such as Jaccard and Bray–Curtis. A Shapiro–Wilk normality test was applied to the data before analyzing the statistical significance of the differences between the experimental groups in alpha diversity with either a Student T-test for normally distributed data or a Wilcoxon Rank Test for the rest. Beta diversity significant differences were determined using PERMANOVA (Anderson, 2001). The phyloseq package, along with fantaxtic (v2.0.1) and vegan (v2.6–4) [46], was used for data analysis and visualization. A p-value of < 0.05 was considered statistically significant in all analyses.

Differentially-abundant species were identified using the DESeq2 (v1.39.8) R package [47]. Only species with a prevalence exceeding 5% in the dataset and a False Discovery Rate (FDR) < 0.05, determined by Likelihood Ratio Test significance tests, were considered statistically significant. Log Fold-Change (LogFC) values represent the relative abundance of a taxon between “Healthy” and “Behavioral Problems” groups. Negative LogFC values suggest higher abundance in the "Healthy" group, while positive values suggest higher abundance in the "Behavioral Problems" group. The DESeq2 model was corrected for relevant confounding variables using the “reduce” parameter within the DESeq function. Differentially-abundant taxa values were normalized using the Variance-Stabilizing Transformation.

### Exploratory statistical analysis and variable preprocessing

Given the complex interplay among the study variables, we first conducted exploratory analyses in SPSS (v28.0) and R (v4.3.2) to select the most relevant variables that potentially influence behavioral features and their relationships with gut microbiota and diet. The preliminary results highlighted the influence of age on both diet and microbiota composition and, accordingly, the population was stratified into two groups: "Childhood" (ages 5–10) and "Adolescence" (ages 11 and older).

To investigate the impact of microbial and dietary factors on behavior, we used the Global SDQ score as the primary outcome measure, considering its overall balance and ability to capture a broad range of behavioral issues (emotional, peer problem, and hyperactivity). Exploratory analysis revealed no substantial differences between clinical and subclinical categories within each age group. Consequently, the population was dichotomized into two groups: "Healthy" individuals (categorized as "Normal" on all SDQ scales) and individuals with "Behavioral Problems" (combining subclinical and clinical categories).

### Potential confounders

To account for potential confounding influences, we selected variables as confounders based on established associations with the outcome, previously reported in the literature, and newly identified in our study population through exploratory analyses. We included the following variables: sex, z-BMI, stress, mode of delivery, exclusive breastfeeding, physical activity, parental education, marital status, bowel habits, and antibiotic intake within a month of sample collection. In the adolescent group, respiratory problems were added as a confounding variable based on its association with behavioral outcomes in exploratory analyses. Age was not added as a confounder because we previously stratified it into childhood or adolescence. To avoid spurious linear relationships, continuous variables, such as z-BMI, stress or physical activity, were stratified into categorical groups following the criteria of each questionnaire. Further information about categorical groups is included in the additional file 1: methods S2.

### Statistical analyses

Since most analyses are related to some degree to the linear or logistic relationship between Global SDQ and other variables, the dataset's adherence to several statistical assumptions was assessed to ensure the validity of our analyses. First, we confirm the dataset's normality using the Shapiro–Wilk test. Second, we evaluated the independence of variable errors by examining autocorrelation with the Durbin-Watson test. Third, for continuous variables, we assumed linearity in their relationship with Global SDQ scores, evaluated through scatter plots. Fourth, we examined multicollinearity using correlation matrices, as high correlations among variables can lead to unreliable estimates. When multicollinearity was identified, we performed a Variable Importance Analysis to select the most relevant variables for distinguishing between experimental groups. Finally, we identified significant outliers through leverage plots and Cook's distance, carefully considering their removal to maintain data integrity while preventing undue influence on our results.

Variable selection and comparisons between the dependent variable and potentially independent variables were performed using parametric tests (Student’s t-test) for continuous variables with normal distribution and non-parametric tests (Mann–Whitney U test and Wilcoxon signed-rank test) when distributions did not follow the normality. The Chi-squared test was used to compare and assess categorical variables. We considered statistical significance at *p*-values < 0.05.

To characterize participants' dietary patterns, we employed a statistical approach combining principal component analysis (PCA) with varimax rotation in IBM SPSS Statistics for Windows (v 27.0). The factorial loadings for each generated dietary pattern are shown in Additional File 1: Table S1. Only components with eigenvalues greater than or equal to 0.4 were considered as those that substantially contribute to that specific dietary pattern.

### Mediation analysis of diet and microbiota

Mediation analysis was conducted to test whether the relationship between diet and Global SDQ score was potentially mediated by specific gut microbiota species. We considered mediation when an independent variable (Exposure to a dietary component) influences a dependent variable (Outcome, the Global SDQ score) through the action of a third variable (Mediator, a specific bacterial species) (Additional file 1: methods S3).

### Classificatory models

To classify individuals into Healthy and Behavioral Problem groups based on the most important predictor variables, we trained a series of Penalized Logistic Regression models. These models were built using a combined dataset of normalized, scaled, and trimmed microbial and dietary information (including nutrients, food groups, dietary patterns, and dietary indices). Penalization in these models reduces the influence of highly correlated variables or those with little classificatory power, based on predefined parameters.

We employed the Elastic Regression Technique [48] as our Penalization Model, implemented using the caret R package (v6.0–94) [49]. To obtain p-values and confidence intervals for predictor importance within this penalized model, we translated the models to the glmnet package (v4.1–8) [50] and utilized the hdi package (v0.1–9) [51] for analysis. The Area Under the Curve (AUC) of the Receiver Operating Characteristic (ROC) was used to determine the classification process of the models. Further information on data pre-processing and model adjustment can be found in Additional file 1: Methods S4.

## Results

### Characteristics of children and adolescent groups

The study included 335 participants: 202 (61%) children aged 5 to 10 years and 133 (39%) adolescents aged 11 to 17 years. Of the children, 88 (44%) exhibited behavioral problems, while 114 (56%) belonged to the healthy group. Among adolescents, 46 (36.4%) had behavioral problems, and 87 (64.6%) were healthy. A small subset of participants (*N* = 14) was diagnosed with neurodevelopmental disorders (e.g., autism, ADHD). Due to the limited sample size, no significant differences were found in analyses involving this group. Consequently, these individuals were included in the overall "behavioral problems" category for the main analyses.

When comparing the features of the categorized population (Table 1), we observed significant differences across several health indicators, including sleep disorders, gastrointestinal disorders, and the DII, which were all higher in the behavioral problems group than in the healthy group.

**Table 1:** Characteristics of the study population categorized by age and behavior

**Age group**	**Overall (*N* = 335)**					p	**Children (5–10 years) *N* = 202 (61%)**				p	**Adolescents (11–17 years) *N* = 133 (39%)**				p
	*Healthy*		*Behavioral problems*				*Healthy*		*Behavioral problems*			*Healthy*		*Behavioral problems*		
	M/N	+ SD/%	M/N	+ SD/%			M/N	+ SD/%	M/N	+ SD/%		M/N	+ SD/%	M/N	+ SD/%	
Age mean ± SD	9.2	2.9	10.1	3.1		0.165	7.6	1.6	7.7	1.6	0.6	12.9	1.7	12.7	1.8	0.40
Sex																
Male *N* (%)	88.0	43.8	73.0	54.5		0.06	48.0	42.1	46.0	52.3	0.158	40.0	46.0	27.0	58.7	0.203
Female *N* (%)	113.0	56.2	45.5	44.6			66.0	57.9	42.0	47.7		47.0	54.0	19.0	41.3	
Parent education																
Basic education *N* (%)	129.0	64.2	77.0	57.5		0.22	71	62.3	51	58.0	0.6	58.0	66.7	26.0	56.5	0.26
High education *N* (%)	72.0	35.8	57.0	42.5			43	37.7	37	42.0		29.0	33.3	20.0	43.5	
Marital status																
Separated/single parent *N* (%)	69	34.3	36	26.9		0.19	39	34.2	23	26.1	0.22	30.0	34.5	13.0	28.3	0.56
Married or couple *N* (%)	132	65.7	98	73.1			75	65.8	65	73.9		57.0	65.5	33.0	71.7	
Delivery mode																
C-section *N* (%)	193.0	85.4	147.0	87.5		0.55	111	86	99	89.2	0.5	87.0	88.0	43.0	77.0	0.06
Vaginal delivery *N* (%)	33.0	14.6	21.0	12.5			18	14	12	10.8		11.0	12.0	13.0	23.0	
Exclusive breastfeeding																
Formula or mixed *N* (%)	83.0	41.3	50.0	37.3		0.50	43.0	37.7	33.0	37.5	1.00	40.0	46.0	17.0	37.0	0.36
Maternal *N* (%)	118.0	58.7	84.0	62.7			71.0	62.3	55.0	62.5		47.0	54.0	29.0	63.0	
Bowel movements (per day)																
Not optimal < or > 3 *N* (%)	40	20.0	35	26.1		0.2	94	85.5	64	72.7	0.12	67.0	77.0	35.0	76.1	1.00
Optimal (1–3 bowels) *N* (%)	161	80.1	99	73.6			20	17.5	24	27.3		20.0	23.0	11.0	23.9	
Intestinal transit (Bristol scale)																
Diarrhea or constipation, *N* (%)	178	88.6	109	81.3		0.08	98	86.0	68	77.3	0.14	80.0	92.0	41.0	89.1	0.75
Optimal transit, *N* (%)	23	11.4	25	18.7			16	14.0	20	22.7		7.0	8.0	5.0	10.9	
Sleep disorders						**0.01**					**0.01**					**0.01**
* Yes N (%)*	13	7	28	21.0			8	7	18	20.5		5.0	5.7	10.0	21.7	
* No N (%)*	188	94	106	79.1			106	93	70	79.5		82.0	94.3	36.0	78.3	
*Respiratory problems*																
*Yes N (%)*	80	45.6	105	57.7		0.19	62.00	70.5	87.0	76.3	0.35	18.0	20.7	18.0	39.1	**0.02**
*No N (%)*	96	54.4	54	42.3			27.00	23.7	26.0	29.5		69.0	79.3	28.0	61.0	
*Gastrointestinal disorders*																
*Yes N (%)*	19	10	25	18.7		**0.015**	13	11.4	18	20.5	0.11	6.0	6.9	7.0	15.2	0.14
*No N (%)*	182	91	109	81.3			101	88.6	70	79.5		81.0	93.1	39.0	84.8	
*Antibiotics*																
*Yes N (%)*	16	8	17	12.7		0.16	10	8.8	14	15.9	0.13	6.0	6.9	3.0	6.5	1.00
*No N (%)*	185	92.0	117	87			104	91.2	74	84.1		81.0	93.1	43.0	93.5	
*Non-antibiotic drugs*																
*Yes N (%)*	53	26	42	31.3		0.39	27	23.7	29	33.0	0.16	26	29.9	13	28.3	1.00
*No N (%)*	148	74	92	68.7			87	76.3	59	67.0		61.0	70.1	33.0	71.7	
*ɣBody mass Index z-score (z-BMI) kg/m2*																
*z-BMI mean* ± *SD*	17.8	0.3	18.0		0.3	0.99	16.9	3.9	16.9	3.0	0.9	19.1	3.6	20.1	3.9	0.9
* Overweight/obesity N (%)*	49	24.1	36		26.9	0.35	35	30.7	25.0	28.4	0.8	73.0	83.9	35.0	76.1	0.4
*Normal or underweight N (%)*	152	75.6	98		73.1		79	69.3	63.0	71.6		14.0	16.1	11.0	23.9	
*Waist circumference (WC)*																
*WC mean* ± *SD*	60.3	1.2	61.3		1.2	0.79	54.74	17.8	58.0	11.2	0.14	67.6	14.5	67.7	14.7	0.1
*Obesity risk N (%)*	44	22	34		25	0.51	29.0	25.4	23.0	26.1	1.0	15.0	17.2	11.0	23.9	0.4
*No obesity risk N (%)*	157	78	100		75		85.0	74.6	65.0	73.9		72.0	82.8	35.0	76.1	
*Inventory of daily stressors perceived by parents (total stress)*																
*Stress N (%)*	49	24	44		33.1	0.08	13	11.4	36	40.9	< 0.001	17.0	19.5	27.0	28.7	**< 0.001**
*No stress N (%)*	153	75.7	89		66.9		101	88.6	52	59.1		70.0	80.5	19.0	41.0	
*Physical activity (APALQ)*																
*mean* + *SD*	12.6	0.2	12.5		0.3	0.87	12.28	2.7	12.1	2.7	0.60	13	3.5	13.3	3.6	0.60
*Sedentary N (%)*	55.0	27.4	38.0		28.4	0.90	35	30.7	24	27.3	0.64	20	23	14	30.4	0.41
*Moderately active or active N (%)*	146.0	72.6	96.0		71.6		79	56.4	64	43.6		67	77	32	69.6	
*Healthy Lifestyle score (HLS)*																
*mean* + *SD*	5.9	0.1	5.7		0.2	0.2	5.9	1.7	5.7	1.7	0.42	5.9	1.6	5.6	1.8	0.42
*Sedentary N (%)*	123.0	61.2	91.0		67.2	0.09	70	61.4	63	71.6	0.2	53.0	60.9	27.0	58.7	0.12
*Moderately active or active N (%)*	78.0	38.8	44.0		32.8		44	38.6	25	28.4		34.0	39.1	19.0	41.3	
*Spanish healthy eating index (SHEI) mean* + *SD*	76.8	9.1	75.9		8.6	0.7	78.1	8.4	76.0	7.1	0.1	75.1	9.7	75.9	10.6	0.1
*Low or Need for improvement*	119.0	59.2	87.0		64.9	0.3	64.0	56.1	59.0	67.0	0.1	55.0	63.2	28.0	61.0	0.9
*Optimal N (%)*	82.0	41.0	47.0		35.1		50.0	43.9	29.0	33.0		32.0	36.8	18.0	39.1	
*Adherence Mediterranean diet (MD) mean* + *SD*	7.0	2.4	6.8		2.3	0.5	7.3	2.2	6.7	2.2	0.10	6.7	2.6	6.5	2.5	0.2
*Low or needs for improvement N(%)*	117	58	81		60	0.68	66	57.9	51	58.0	1.00	51	58.6	30	65.2	0.58
*Optimal,N (%)*	84	42	53		40		48	42.1	37	42.0		36	41.4	16	34.8	
*Diet Inflammatory Index score (DII)*	0.1	1.6	0.4		1.8	**0.04**	0.1	1.5	0.5	1.7	0.1	0.0	1.7	0.4	1.9	0.1
*Pro-Inflammatory N (%)*	38.0	18.9	37.0		27.6	0.2	21.0	18.4	26.0	29.5	0.1	17.0	19.5	11.0	23.9	0.8
*Neutral, N (%)*	107.0	53.2	63.0		47.0		64.0	56.1	40.0	45.5		43.0	49.4	23.0	50.0	
*Anti-Inflammatory N (%)*	56.0	27.9	34.0		25.4		29.0	25.4	22.0	25.0		27.0	31.0	12.0	26.1	

Considering as “Healthy” the subjects without behavioral problems measured by the SDQ.*Data are expressed as mean (SD) or percentage (N). Statistical tests: Mann–Whitney U, Kruskal–Wallis, and Student`s t-test were used for quantitative variables and Chi-squared for qualitative variables. Significant differences are in bold (*p* < 0.05). ɣBMI z-scores were used to classify participants with obesity/overweight and normal weight/underweight following WHO international cut-offs

Further analysis by age group demonstrated a strong association between sleep disorders and both children and adolescents with behavioral problems, whereas increased respiratory disorders were only associated with adolescents (Table 2). Therefore, stress in children and adolescents, along with respiratory disorders in the adolescent population, were included as confounders in subsequent analyses. No other assessed confounders had a significant effect.

**Table 2:** Association between hebavioral problems and other personal features and lifestyle factors

**Overall population**	**Exp(β) (95% CI)**	***p*-value**	**q-value**
Daily perceived stressors	1.682 (0.96, 2.4)	< 0.001	< 0.001
Sleep disorders	3.39 (1.58, 7.30)	0.01	0.02

**Childhood population**	**Exp(β) (95% CI)**	***p*-value**	**q-value**
Daily stressors perceived by parents	1.682 (0.96, 2.4)	< 0.001	< 0.001

**Adolescent population**	**Exp(β) (95% CI)**	***p*-value**	**q-value**
Respiratory problems	2.7 1.14, 6.6)	0.03	0.04
Daily perceived stressors	1.76 (0.97, 2.56)	0.01	0.01

Exp (β) (95% CI) = Exponential of the coefficient β, representing an odds ratio

*P* Not adjusted for covariables (*p* < 0.05). Only variables associated with behavioral problems are shown

### Alpha diversity, but not beta diversity, differs in subjects with behavioral problems compared to healthy individuals

We investigated potential associations between gut microbiota differences and behavioral problems separately in the two age groups, given that dietary intakes, which significantly influence the gut microbiota, varied substantially between children and adolescents.

**Fig. 2:**
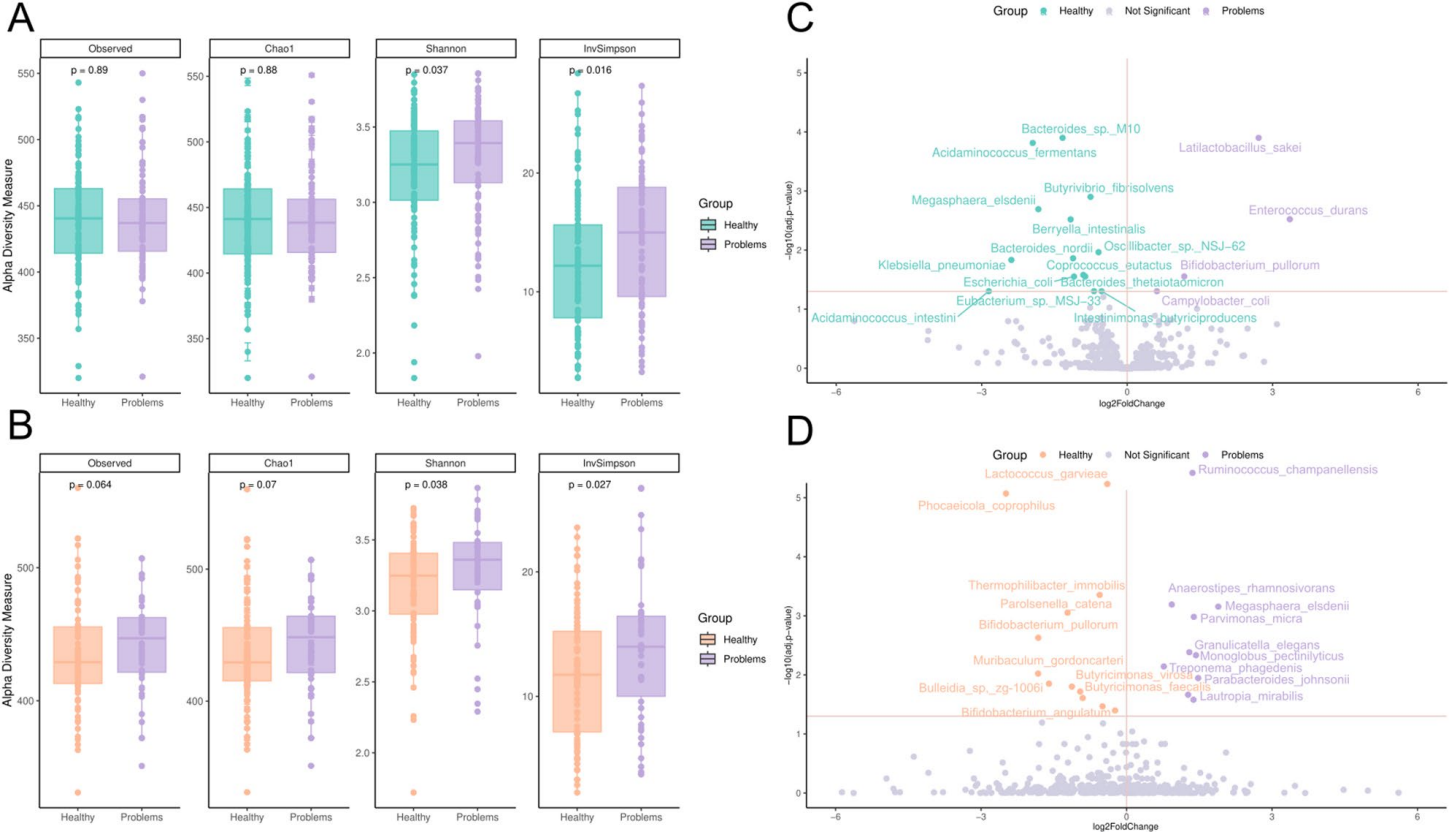
Metataxonomic analysis of the gut microbiome in children and adolescents. **A** Alpha diversity indices (Observed abundance, Chao1, Shannon and Inverse Simpson) in children and their statistical significance based on the Wilcoxon test (*p* < 0.05) corrected for confounders. **B** Alpha diversity indices (Observed abundance, Chao1, Shannon and Inverse Simpson) in adolescents and their statistical significance based on the Wilcoxon test (*p* < 0.05) corrected for confounders. **C** Differentially abundant bacteria between “Healthy” and “Behavioral Problems” groups in children. **D** Differentially abundant bacteria between “Healthy” and “Behavioral Problems” groups in adolescents

Regarding alpha diversity, we observed increased alpha diversity, as measured by the inverse Simpson index, associated with behavioral problems in both age groups after pair comparisons adjusted by confounders (Fig. 2A and B). A similar significant trend was observed for the inverse of the Simpson index using regression analysis, after adjusting for confounders, in both children and adolescents (Additional file 1: Table S2). Conversely, we found no associations between beta diversity and global behavioral problems (Additional file 1: Table S3).

### Gut bacterial species differ in subjects with behavioral problems

Analysis of the gut microbiome revealed significant differences in the abundance of 19 bacterial species between children with behavioral problems and healthy children. Specifically, children with behavioral problems had a higher abundance of *Enterococcus durans, Campylobacter coli, Lactobacillus sakei*, and *Bifidobacterium pullorum*, while healthy children had higher levels of *Clostridium autoethanogenum*, *Acidaminococcus* spp., *Bacteroides* spp., *Coprococcus eutactus, Butyrivibrio fibrisolvens* and *Eubacterium* sp. MSJ 33, among other species (see Table 3 and Fig. 2C).

**Table 3:** Differential abundance of bacterial species in children with behavioral problems

**Bacterial species**	Log2FC*	**P**	q*
*Bacteroides nordii*	−1.12	2.12E + 02	1.38E + 04
*Bacteroides* sp. M10	−1.33	5.82E-01	1.26E + 02
*Bacteroides thetaiotaomicron*	−0.86	5.67E + 02	2.80E + 04
*Campylobacter coli*	0.61	1.26E + 03	4.97E + 04
*Klebsiella pneumoniae*	−2.39	2.49E + 02	1.47E + 04
*Escherichia coli*	−1.10	6.49E + 02	2.81E + 04
*Berryella intestinalis*	−1.17	3.73E + 01	3.03E + 03
*Bifidobacterium pullorum*	1.18	6.03E + 02	2.80E + 04
*Megasphaera elsdenii*	−1.83	1.88E + 01	2.03E + 03
*Acidaminococcus fermentans*	−1.95	9.47E-01	1.54E + 02
*Acidaminococcus intestini*	−2.85	1.45E + 03	4.97E + 04
*Enterococcus durans*	3.35	3.24E + 01	3.01E + 03
*Latilactobacillus sakei*	2.71	4.35E-01	1.26E + 02
*Eubacterium* sp. MSJ-33	−0.68	1.35E + 03	4.97E + 04
*Intestinimonas butyriciproducens*	−0.53	1.39E + 03	4.97E + 04
*Clostridium autoethanogenum*	−25.58	1.85E-12	1.20E-09
*Oscillibacter* sp. NSJ-62	−0.59	1.51E + 02	1.09E + 04
*Butyrivibrio fibrisolvens*	−0.76	9.68E + 00	1.26E + 03
*Coprococcus eutactus*	−0.91	4.88E + 02	2.64E + 04

Species associated with behavioral problems

^*^Log2FC = Log2 fold-change representing the relative abundance difference in the behavioral problems group compared with the healthy group; positive values indicate greater abundance in the behavioral problems group and negative values indicate greater abundance in the healthy group. *p* < 0.05. q is FDR-adjusted for age, sex, z-BMI, stress, antibiotics, and other medications

Among adolescents, significant differences were observed in the abundance of 22 bacterial species between those with behavioral problems and those who were healthy. Specifically, the gut microbiota of the behavioral problem group was enriched in taxa such as *Pyramidobacter psicolens*, *Parabacteroides johnsonii*, *Lautropia mirabilis,* and *Anaerostipes rhamnosivorans,* while *Muribaculum gordoncarteri*, *Butyricimonas* spp., *Phocaeicola coprophilus*, and *Bifidobacterium* spp., were more abundant in the healthy group (see Table 4 and Fig. 2D).

**Table 4:** Differential abundance of bacterial species in adolescents with behavioral problems

**Bacterial species**	Log2FC*	**p**	q*
*Pyramidobacter piscolens*	1.21	1.86E-03	4.26E-02
*Treponema phagedenis*	1.00	3.88E-04	2.04E-02
*Muribaculum gordoncarteri*	−1.80	2.62E-04	1.02E-02
*Butyricimonas faecalis*	−1.07	6.58E-04	1.95E-02
*Butyricimonas virosa*	−1.51	1.09E-03	4.46E-02
*Parabacteroides johnsonii*	1.54	7.12E-04	1.99E-02
*Phocaeicola coprophilus*	−2.26	1.53E-04	1.15E-02
*Lautropia mirabilis*	1.59	4.00E-04	2.04E-02
*Parafannyhessea umbonata*	−1.05	6.25E-04	1.95E-02
*Thermophilibacter immobilis*	−1.55	8.86E-04	4.02E-02
*Parolsenella catena*	−1.04	5.81E-05	5.94E-03
*Bifidobacterium pseudolongum*	−0.20	1.12E-03	2.92E-02
*Bifidobacterium pullorum*	−1.61	1.65E-03	4.95E-02
*Bifidobacterium angulatum*	−0.12	1.51E-03	3.62E-02
*Megasphaera elsdenii*	2.61	1.69E-04	1.15E-02
*Parvimonas micra*	1.57	3.08E + 09	1.94E-03
*Granulicatella elegans*	1.40	1.26E-04	6.32E-03
*Lactococcus garvieae*	−0.23	2.31E + 07	5.81E + 09
*Bulleidia sp, zg-1006*	−1.50	3.27E-04	1.10E-02
*Monoglobus pectinilyticus*	1.53	1.59E-03	4.95E-02
*Ruminococcus champanellensis*	1.64	2.36E-06	4.82E-04
*Anaerostipes rhamnosivorans*	1.08	1.54E-05	2.10E-03

Species associated with behavioral problems. *Note: Log2FC = Log2 fold-change representing the relative abundance difference in the behavioral problems group compared with the healthy group; positive values indicate greater abundance in the behavioral problems group and negative values indicate greater abundance in the healthy group. *p* < 0.05. q is FDR-adjusted for age, sex, z-BMI, stress, respiratory disorders, antibiotics and other medications

### Differences in the adherence to SENC dietary guidelines among groups

Analysis of adherence to the SENC dietary guidelines showed that children with behavioral problems exhibited significantly higher iodine, energy, and cholesterol intake and a lower intake of linoleic acid compared with nutrient recommendations. No differences in nutrient intake were found in the adolescent population. Analysis of food group recommendations revealed that children with behavioral problems consumed significantly higher amounts of processed food, milk and dairy products, and processed meat, while adolescents with behavioral problems consumed fewer fruits and vegetables and more fish than recommended (Table 5).

**Table 5:** Percentage of food group consumption (servings per day/week) and nutrients (g/day) according to participants' compliance with the recommended dietary intakes (RDI) established by dietary guidelines

Nutrients and food groups	**Children (5–10 years) *N* = 202 (61%)**						p	**Adolescents (11–17 years) *N* = 133 (39%)**						p
	**Healthy**			**Behavioral problems**				**Healthy**			**Behavioral problems**			
	Comply RDI *	Over RDI*	Below RDI*	Comply RDI	Over RDI	Below RDI		Comply RDI	Over RDI	Below RDI	Comply RDI	Over RDI	Below RDI	
Energy (kcal)	48.2	5.3	46.5	58	16	26.1	**0.00**	40.2	11.5	48.3	45.7	17.4	37	0.40
Protein (g)	63.2	36.8	0	64.8	35.2	0	0.96	55.2	44.8	0	67.4	32.6	0	0.39
Total fiber (g)	73.7	NA	49.1	71.6	NA	28.4	0.75	83.9	NA	16.1	80.4	NA	19.6	0.64
Fats (g)	50.9	49.1	0	61.4	38.6	0	0.16	57.5	42.5	0	67.4	32.6	0	0.35
Linolenic acid (g)	40	NA	60	55.3	NA	44.7	0.05	57.5	NA	42.5	58.7	NA	41.3	1.00
Linoleic acid (g)	58	NA	42	42	NA	58	**0.03**	73.6	NA	26.4	84.8	NA	15.2	0.14
DHA + EPA (mg)	74.6	NA	25.4	70.5	NA	29.5	0.53	85.1	NA	14.9	91.3	NA	8.7	0.42
SFA (g)	23.7	76.3	NA	23.9	76.1	NA	1.00	25.3	74.7	NA	28.3	71.7	NA	0.84
PUFAS (g)	14.9	NA	85.1	18.2	NA	81.8	0.57	78	NA	21.8	84.8	NA	15.2	0.36
MUFAS (g)	56.1	NA	44	51.1	NA	48.9	0.57	66.7	NA	33.3	56.5	NA	43.5	0.26
Cholesterol (mg)	63.2	36.8	NA	38.6	61.4	NA	**< 0.00**	37.9	62.1	NA	28.3	71.7	NA	0.40
Iodine (µg)	34.2	NA	65.8	50	NA	50	**0.02**	58.6	NA	41	76.1	NA	24	0.06
Sodium (mg)	62.3	37.7	NA	48.9	51.1	NA	0.06	23	77	NA	11	89.1	NA	0.11
Potassium (mg)	84.2	NA	15.8	92	NA	8	0.13	79.3	NA	20.7	84.8	NA	15.2	0.49
Calcium(mg)	36.8	NA	63.2	50	NA	50	0.06	35.6	NA	64.4	32.6	NA	67.4	0.85
Total folate (B9) (µg)	75.4	NA	24.6	78.4	NA	21.6	0.74	74.7	NA	25.3	69.6	NA	30.4	0.54
Vitamin A (µg)	81.6	NA	18.4	85.2	NA	14.8	0.57	85.1	NA	15	91.3	NA	8.7	0.42
Vitamin D (µg)	13.2	NA	86.8	19.3	NA	80.7	0.25	23	NA	77	30.4	NA	69.6	0.41
Vitamin E (mg)	71.1	NA	28.9	78.4	NA	21.6	0.26	65.5	NA	34.5	50	NA	50	0.10
Total polyphenols (mg)	60.5	NA	39.5	69.3	NA	30.7	0.24	73.6	NA	26.4	71.7	NA	28.3	0.84
Sugar Beverage (ser/day)	57	43	NA	48.9	51.1	NA	0.25	51.7	48.3	NA	56.5	43.5	NA	0.60
Fruit and vegetables (ser/day)	46.5	NA	53.5	44.3	NA	55.7	0.86	58.6	NA	41.4	37	NA	63	**0.02**
Legumes (ser/day)	58.8	15.8	25.4	51.1	20.5	28.4	0.52	47.1	31	21.8	43.5	43.5	13	0.27
Nuts and oil olive (ser/day)	65.8	NA	34.2	73.9	NA	26.1	0.22	62.1	NA	37.9	67.4	NA	32.6	0.53
Processed food (ser/day)	23.7	76.3	NA	11.4	88.6	NA	**0.03**	25.3	74.7	NA	19.6	80.6	NA	0.46
Pastries (ser/day)	10	90	NA	5.7	94.3	NA	0.30	13.8	86.2	NA	6.5	93.5	NA	0.21
Milk and diary products (ser/day)	94.7	5.3	0	85.2	14.8	0	**0.02**	90	10	0	89	11	0	0.76
Refined cereals (ser/day)	22	78	NA	19.3	80.7	NA	0.65	24.1	75.9	NA	26.1	73.9	NA	0.80
Whole grain cereals (ser/day)	2	NA	98	3.4	NA	96.4	0.45	4.6		95.4	6.5		93.5	0.64
Fish(ser/day)	39.5	41.2	19.3	26.1	52.3	21.6	0.13	24.1	59.8	16.1	32.6	34.8	32.6	**0.02**
Red meat (ser/day)	26.3	73.7	NA	17	83	NA	0.12	17.2	82.8	NA	13	87	NA	0.53
White meat (ser/day)	93.9	6.1	NA	90.1	9.1	NA	0.43	94.3	5.7	NA	89.1	10.9	NA	0.29
Processed meat (ser/day)	15.8	84.2	NA	4.5	95.5	NA	**0.01**	3.4	96.6	NA	6.5	93.5	NA	0.42

Food group recommendations are expressed per serving and per day (ser/day) or per week (ser/wk). Nutrient recommendations are expressed per gram, milligram or microgram per day (g, mg or µg)

^*^ RDI: Recommendation dietary intakes. RDIs are expressed in percentage (%) of participants who comply or do not comply by over intake or below intake recommendation. Statistical differences were calculated using the Chi-squared test. Differences are shown in bold (*p* < 0.05

### Identified patterns and differences in adherence between healthy and behavioral problem group

**Fig. 3:**
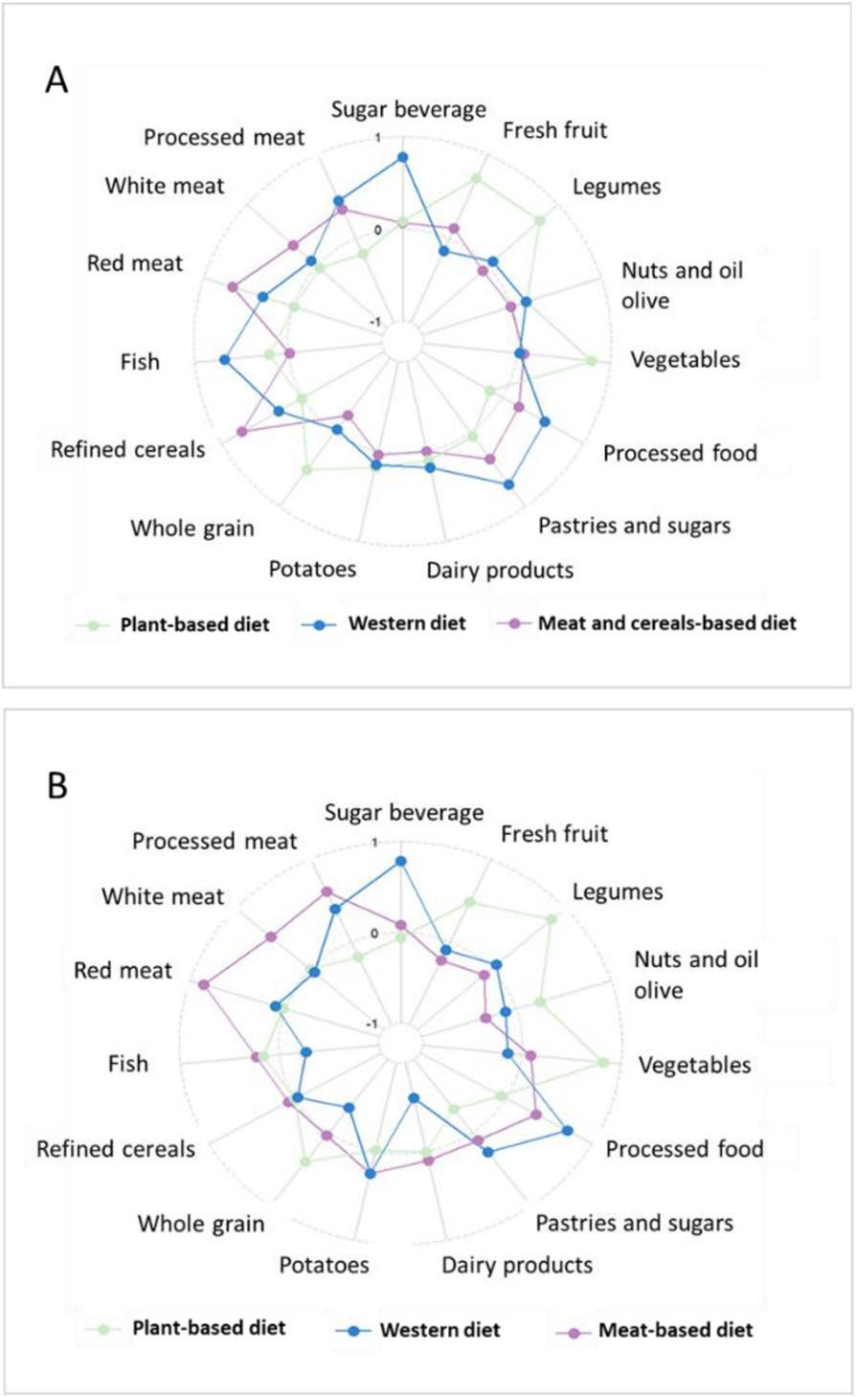
Dietary Patterns in children and adolescents. Radar chart showing dietary patterns extracted from principal component analysis of 15 food groups in (**A**) children and (**B**) adolescents

Three distinct dietary patterns emerged, exhibiting similar trends for both age groups: a “Plant-based diet”, rich in fruits, legumes, vegetables, and whole grains; a "Western diet”, rich in pastries, sugars, sugar beverages, and processed foods (and fish in adolescents); and a “Meat and Cereals based diet” for children or a “Meat-based diet” for adolescents (Fig. 3). Adherence to the Western dietary pattern was significantly higher in children with behavioral problems than in healthy peers. Although not significant, a similar trend was observed in adolescents. No significant differences were found for the other two dietary patterns in either age group (Table 6).

**Table 6:** Differences in dietary pattern adherence between children and adolescents with and without behavioral problems

**Dietary patterns**	**Children (5–10 years) *N* = 202 (61%)**					**Adolescents (11–17 years) *N* = 133 (39%)**				
	Healthy		Behavioral problems		*p*	Healthy		Behavioral problems		*p*
	*N*	(%)	*N*	(%)		*N*	(%)	*N*	(%)	
Plant-based pattern										
Low adherence	38.0	33.3	29.0	33.0	1.0	25.0	28.7	18.0	39.1	0.40
Medium	38.0	33.3	30.0	34.1		33.0	37.9	13.0	28.3	
High adherence	38.0	33.3	29.0	33.0		29.0	33.3	15.0	32.6	
Western pattern										
Low adherence	47.0	41.0	20.0	22.5	**0.02**	35.0	40.2	10.0	21.7	0.07
Medium	35.0	31.0	33.0	37.5		28.0	32.2	16.0	34.8	
High adherence	32.0	28.0	35.0	40.0		24.0	27.6	20.0	43.5	
Meat pattern										
Low adherence	44.0	38.6	23.0	26.1	0.1	30.0	34.5	14.0	30.4	0.1
Medium	38.0	33.3	42.0	47.7		34.0	39.1	12.0	26.1	
High adherence	32.0	28.1	23.0	26.1		23.0	26.4	20.0	43.5	

^*^Data are expressed as percentage (*N*). Statistical differences were calculated using the Chi-squared test. Differences are shown in bold (*p* < 0.05)

### Behavioral problems are consistently associated with a Western dietary pattern and deficits in the intake of related nutrients and food groups

When analyzing nutrient and food group intakes adjusted for energy, children with behavioral problems had lower intakes of fresh fruit, total fiber, soluble fiber, insoluble fiber, and vitamin B9, and tended to have a higher intake of fructose. Adolescents with behavioral problems had higher intake of fish and docosahexaenoic acid (DHA), but lower intake of legumes, nuts, olive oil, and vegetables (Additional file 1: Table S4 and S5).

We used logistic regression models to investigate the association between behavioral problems and various dietary factors, including 15 food groups, 49 nutrients, three dietary indices, and adherence to the three distinct dietary patterns. In both populations, we found that behavioral problems were associated with adherence to the Western dietary pattern, even after adjusting for confounders. Also, lower consumption of soluble fiber and vitamin B9 was associated with behavioral problems in children, and lower consumption of legumes, nuts, and olive oil was associated with behavioral problems in adolescents after adjusting for potential confounders. Finally, an increased intake of fish and DHA was associated with behavioral problems in adolescents (Table 7).

**Table 7:** Associations of nutrients, food groups and dietary patterns with behavioral problems

**Childhood population**	**Exp(β) (95% CI)**	**p**	**padj**
Soluble fiber(g)	0.33 (0.10, 0.61)	0.01	0.02
Vitamin B9 or folate(µg)	0.28 (0.09, 0.82)	0.02	0.07*
Western dietary pattern	1.57 (1.15, 2.10)	0.002	0.02

**Adolescents population**	**Exp(β) (95% CI)**	**p**	**padj**
Docohexaenoic acid (DHA) (mg)	2.34 (1.09, 4.74)	0.03	0.02
Legumes (g)	0.52 (0.30, 0.95)	0.04	0.04
Nuts and oil olive (g)	0.67 (0.47, 0.74)	0.03	0.01
Fish (g)	2.27 (1.16, 4.42)	0.02	0.01
Western dietary pattern	1.51 (1.15, 2.09)	0.04	0.04

Exp(β) (95% CI) Exponential of the coefficient B. It represents odds ratio. p: Model not adjusted by covariables. padj: Model adjusted by sex, z-BMI, parent education, bowels, APALQ, stress level, and medication and antibiotics intake. Only nutritional variables showing significant differences are included (*p* < 0.05)

^*^ (*p* < 0.1)

### Diet and behavioral associations are mediated by specific bacterial taxa

**Fig. 4:**
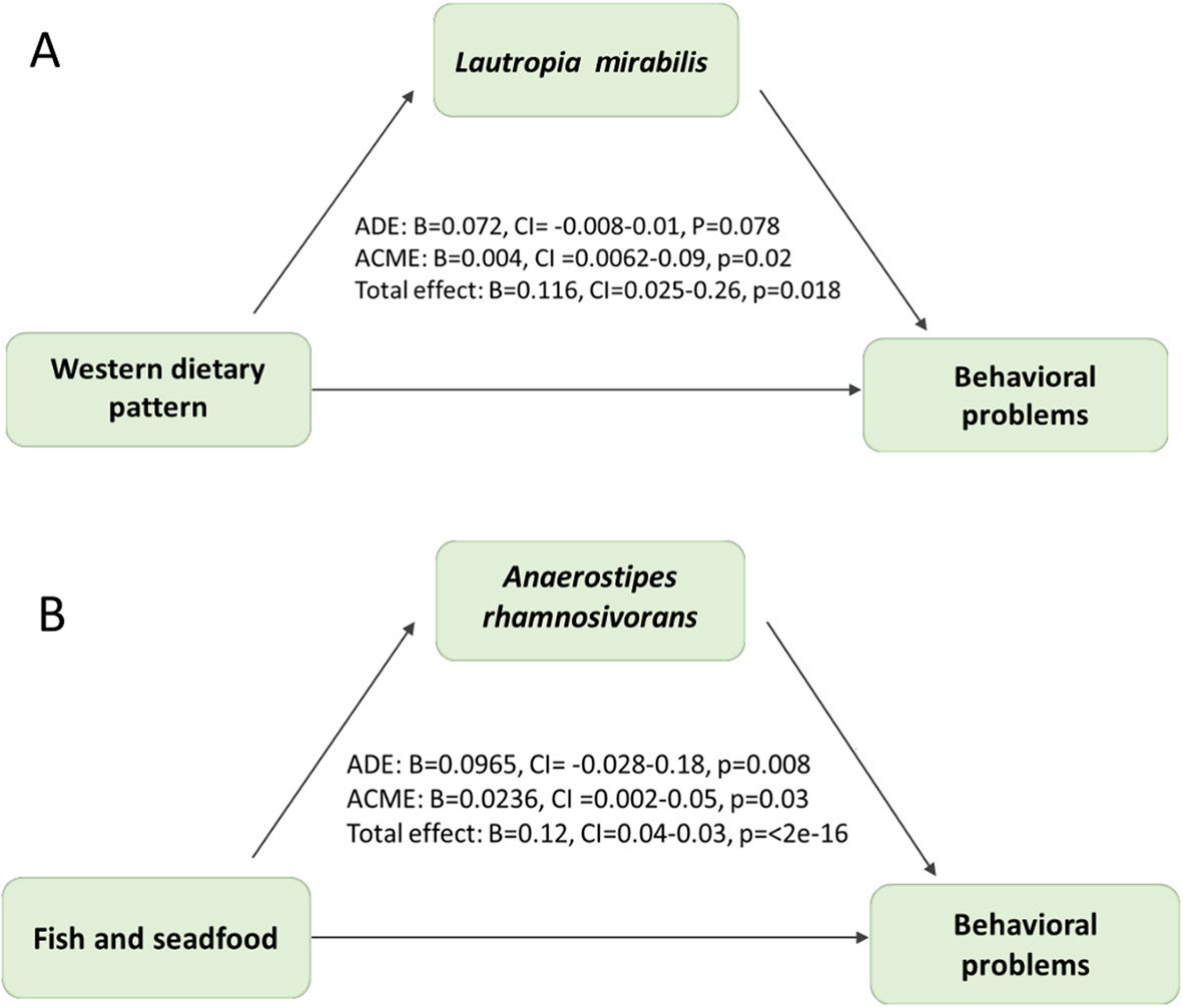
Mediation analysis in adolescents. **A** The mediating effect of *Lautropia mirabilis* on the relationship between adherence to a Western diet and behavioral problems, considering both the direct effect (ADE) of the Western diet and its interaction with the indirect effect (ACME) mediated through *Lautropia mirabilis*. **B** The mediating effect of *Anaerostipes rhamnosivorans* on the relationship between fish consumption and behavioral problems, considering both the direct effect (ADE) of fish consumption and its interaction with the indirect effect (ACME) mediated through *Anaerostipes rhamnosivorans*

Analysis of the gut microbiota as a mediator of the diet-behavior relationship revealed significant effects only in adolescents (Additional file 1: Table S6). Specifically, after adjusting for confounders, the association between a Western diet and behavioral problems was significantly mediated by the abundance of *Lautropia mirabilis* (see indirect effect, Fig. 4A). In contrast, the association between fish intake and behavioral problems was significantly mediated by the abundance of *Anaerostipes rhamnosivorans* (see indirect effect, Fig. 4B). No mediating effects of the gut microbiome were found to explain the association between behavioral problems and the inverse Simpson diversity index in either children or adolescents. Furthermore, no significant mediating effects were observed for other tested gut microbiota or dietary variables.

### Classificatory models of behavior integrating dietary and microbiome data

To classify behavioral outcomes, we developed models integrating dietary and microbiota data. The main bacterial species marked as the most relevant predictors included *B. fibrisolvens* and *C. coli* in the children group, while *P.catena* and *R. champanellensi*s appeared in the adolescents group. Relevant contributions of the remaining variables for each one of the models are shown in additional file 1: Table S7 for children and additional file 1: Table S13 for adolescents.

Regarding the selection of dietary factors, in children, the most discriminant dietary factors were fructose and vitamin B9 at the nutrient level, and pastries, processed food, white meat, and processed meat at the food group level (additional file 1: Table S8-S10). In adolescents, fiber (both total and insoluble), DHA, and eicosapentaenoic acid (EPA) were identified as highly discriminative. These findings partly support the results found for food groups, where legumes and fish (sources of fiber and DHA, respectively) were the more relevant items associated with behavioral problems (additional file 1: Table S14 -S16).

**Fig. 5:**
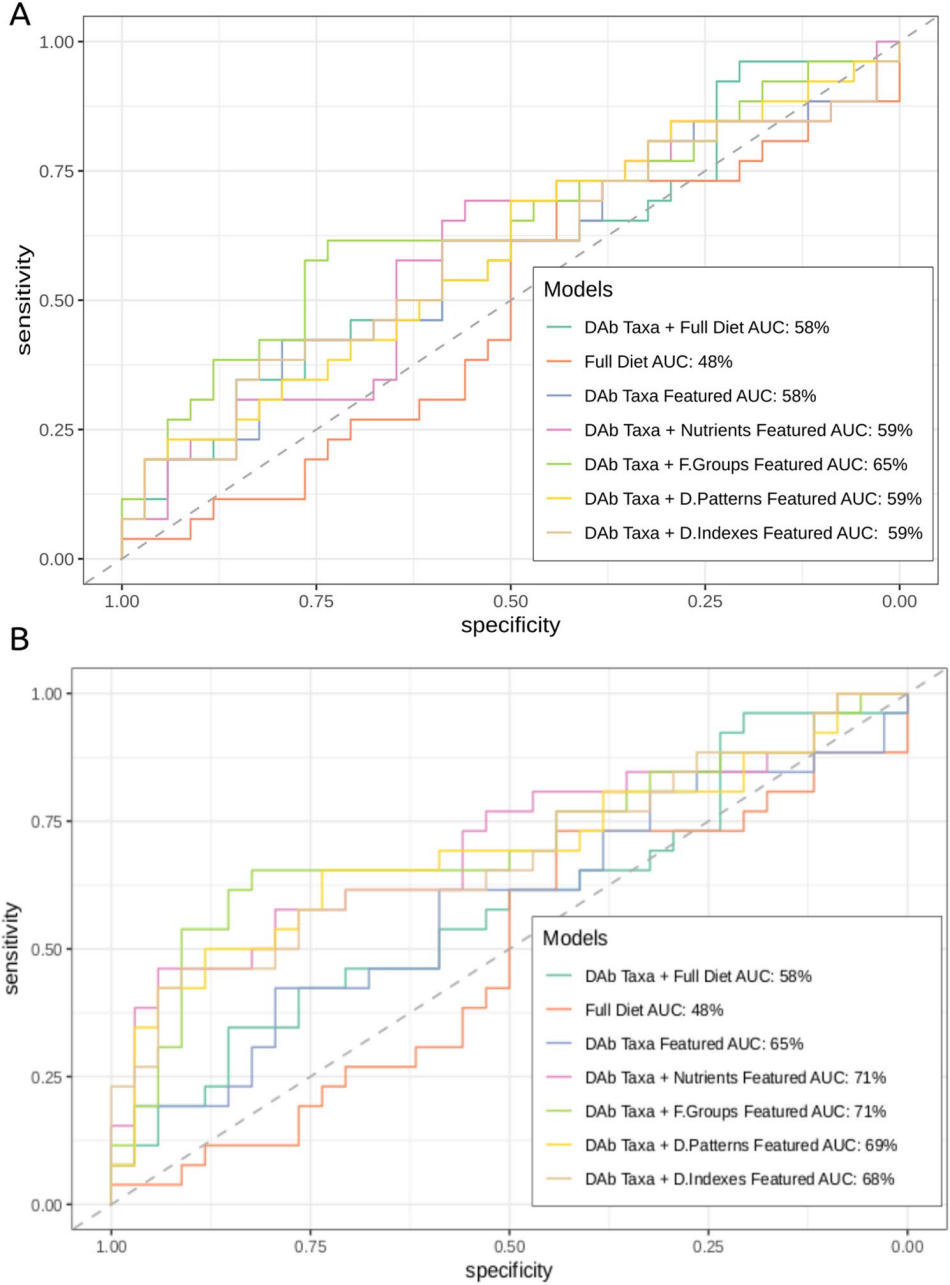
Receiving operating characteristic (ROC) curves of diet and microbiota penalized logistic regression models. Each curve corresponds to a different combination of dietary elements and meta-taxonomical items, considering the area under the curve (AUC) to address the discriminant power of the model between “Healthy” and “Behavioral Problems” groups. Models are based on the following data combinations: DAb Taxa (differentially abundant bacterial species); Full Diet (standalone combination of diet items, including nutrients, food groups, dietary patterns and dietary indexes); DAb Taxa + Nutrients featured (combination of feature-selected bacterial species and nutrients); DAb Taxa + F.Groups featured (combination of feature-selected bacterial species and food groups); DAb Taxa + D.Patterns featured (combination of feature-selected bacterial species and dietary patterns); DAb Taxa + D.Indexes featured (combination of feature-selected bacterial species and dietary indices). **A** ROC curves for children using previously-selected dietary and taxonomical elements through feature selection. **B** ROC curves for adolescents using previously-selected dietary and taxonomical elements through feature selection

A total of fourteen models (seven for each age group) were trained using a different combination of bacterial and dietary data in each to assess their potential as classifiers (Fig. 5). Models utilizing full dietary information alone demonstrated the lowest classificatory performance (Fig. 5A, B), with AUCs of 48% in both children and adolescents, aligning with the estimated p-values associated with the dietary components. By contrast, models using differentially abundant bacteria alone exhibited higher classificatory performance, achieving AUCs of 58% in children and 65% in adolescents (Fig. 5A, B). Notably, combining differentially abundant bacteria with all dietary features did not improve classificatory performance. However, models combining differentially abundant bacteria with individual dietary components (nutrients, food groups) achieved the highest AUC values, reaching 65% in children and 71% in adolescents (Fig. 5A, B). Relevant contributions of the remaining variables for each one of the models are listed in Additional file 1: Table S7-S12 for children and Additional file 1: Table S13-S18 for adolescents.

## Discussion

The present study provides new insights into the associations between dietary patterns, gut microbiota, and behavioral problems in children and adolescents. Our findings specifically support an association between a Western dietary pattern and behavioral problems across both age groups, but with particularities regarding the nutrients and foods involved at each age. Gut microbiota data also improved the classification accuracy of mathematical models for identifying individuals with behavioral problems compared with dietary data alone, suggesting a role for gut microbes as potential biomarkers.

Utilizing in-depth dietary analysis and shotgun metagenomics, our findings reveal that children and adolescents with behavioral problems exhibit increased alpha diversity within their gut microbiota. While higher alpha diversity is often associated with a healthy microbiota and improved mental health outcomes, such as temperament [11] and behavior [19], the quality and function of the microbial species within the gut ecosystem can be equally crucial for neurocognitive and neuropsychiatric outcomes. Consistent with our findings, studies have linked greater alpha diversity to major depression in adults [52] and autism in older children [53]. However, no studies focus attention on the influence of diet on microbial diversity in children and adolescents with behavioral outcomes. Related to this, a recent study found that consuming processed foods in early life may increase alpha diversity in a detrimental way. Therefore, the consumption of this type of food could be a key factor in shaping the microbiota pattern in children with behavioral problems [54].

We observed increased levels of *C. coli* and *E. durans* in children with behavioral problems. *Campylobacter* spp. is one of the four major global causes of diarrheal disease [55], and its presence in the intestinal microbiota has been linked to child growth and neurodevelopmental issues [56]. Similarly, preclinical studies show that oral ingestion of *Enterococcus* species can induce depression-like behavior and neuroinflammation [57]. While we also found increased levels of beneficial bacteria such as *Bifidobacterium* and *Latilactobacillus* (*L. sakei* and *B. pollurum*) in this group, these are likely linked to dietary factors, particularly the consumption of fresh and processed meats [58], and are not necessarily linked to neurodevelopmental disorders in children [59, 60].

Among the bacterial species found to be more abundant in healthy children than in peers with behavioral problems, notable examples include *B. nordii*, *B. thetaiotaomicron*, *C. eutactus* and *I. butyriciproducens*, which have been positively linked to normal cognitive functioning [14, 61, 62]. Notably, *C. euctatus* was also more abundant in healthy children than in peers with depressive and anxious behavior in a recent cross-sectional study [62]. *Bacteroides* species and other commensals (e.g., *I. butyriciproducens*) are specialized in utilizing dietary fibers to produce SCFAs, which can strengthen the gut and brain barrier, reduce inflammation, and exert neuroprotective effects [63–66]. Furthermore, a preclinical study demonstrated that *C. eutactus* can produce serotonin from tryptophan, potentially influencing neuronal plasticity and glial activity in the prefrontal cortex [67], which could also be of help for children with mental health issues, where serotonin production is often impaired [68].

Notably, children with behavioral problems exhibited a strong association with a Western dietary pattern, characterized by a high intake of cholesterol- and energy-rich foods such as processed foods or meat, and a low intake of foods rich in soluble or insoluble fiber such as fruits. This was in line with a lack of full compliance with dietary recommendations. These children also showed lower intake of vitamin B9. A recent in vitro study [69] demonstrated that folic acid deficiency negatively impacts gut microbiota composition and SFCA production. Furthermore, research supports the idea that altered folate metabolism could contribute to the development of ASD, suggesting a potential link between vitamin B9 deficiency and gut health in children with behavioral issues [70].

Consistent with our findings, recent cross-sectional studies in preschoolers and school-age children have described a positive association between Western dietary patterns and hyperactivity and inattention behavior [71–73]. Furthermore, a deficiency in dietary fiber consumption coupled with a Western diet might underlie poor gut immune homeostasis and contribute to neurological disease development [74]. Conversely, a cross-sectional study in children and adolescents demonstrated that diets rich in fiber, vitamins and minerals, and low in saturated fats and cholesterol, are associated with improved cognitive function [75].

In adolescents, a notable finding of our study was the significant association between decreased levels of *Bifidobacterium* and *Butyricimonas* species and behavioral problems. This aligns with previous findings in adults, where a decreased abundance of the genus *Butyricimonas* was observed in patients with cognitive impairment [76]. Notably, several *Bifidobacterium* species identified in our study, including *B. angulatum* and *B. pseudolongum*, are known to produce GABA, an inhibitory neurotransmitter crucial for cognition, behavior, anxiety, and stress response [77–79]. Furthermore, *Butyricimonas faecalis* produces propionate and *Butyricimonas virosa* produces butyrate, which are SCFAs known to reduce BBB permeability and exert anti-inflammatory effects in the brain in mice [80].

Similar to children, we found associations between adherence to a Western diet and behavioral problems in adolescents. This is consistent with observations of reduced consumption of healthy foods, such as vegetables, legumes, nuts, and olive oil, among adolescents with behavioral issues [81]. These foods are rich in fiber and anti-inflammatory compounds, which are typically under-represented in Western diets. Recent research has demonstrated associations between Western dietary patterns and depressive behavior in adolescents [82].

Mediation analysis revealed that the association between behavioral problems and a Western diet in adolescents was partially mediated by *L. mirabilis*, a Gram-negative, facultatively anaerobic bacterium commonly found in the human oral cavity and upper respiratory tract [83]. *L. mirabilis* has been previously associated with infections in immunocompromised subjects and cystic fibrosis [84, 85]. Unexpectedly, we also found an association between increased fish intake (and consequently higher DHA levels) and behavioral problems in adolescents. While DHA (an omega-3 fatty acid) is crucial for brain development and cognition [86], our analyses suggest that this association might be mediated (at least partly) by *A. rhamnosivorans,* a bacterium linked to respiratory conditions and inflammation [87]. A post-hoc analysis ruled out a significant effect of respiratory problems on this mediation, further supporting the role of *A. rhamnosivorans* as a diet-based mediator of the observed associations.

While fish is generally considered beneficial due to its omega-3 fatty acid content, it can also be a source of harmful substances, such as heavy metals, trimethylamine-N-oxide (TMAO), dioxins, polychlorinated biphenyls, and microplastic particles. These contaminants can disrupt gut microbiota balance (dysbiosis) and permeability, potentially impacting behavior [88, 89]. TMAO, produced by gut microbiota from dietary precursors such as choline, L-carnitine, and phosphatidylcholine, has been linked to increased severity of ASD symptoms in children [90], and may contribute to neuroinflammation and cognitive dysfunction in rodents [91, 92]. Although *A. rhamnosivorans* typically produces butyrate from fiber, in a low-fiber environment, it might contribute to the production of other metabolites from fish that could negatively impact the gut-brain axis, immune function, and behavior [91]. Further mechanistic studies are crucial to fully understand the possible role of *A. rhamnosivorans* in mediating the relationship between fish intake and behavioral issues.

Our findings suggest that nut and olive oil consumption may protect against behavioral problems in adolescents. Walnuts are rich in alpha-linolenic acid, a short-chain omega-3 fatty acid involved in brain development [86], and may benefit cognitive development. Moreover, nut intake can modulate the gut microbiota, increasing the abundance of bacteria contributing to butyrate production, such as *Bifidobacterium* species [21, 93], associated with improved cognitive abilities in adolescents [75, 94]. Preclinical and clinical trials in adults have reported that olive oil, rich in monounsaturated fatty acids and phenolic compounds, has positive effects on the gut microbiota by reducing pathogens and increasing SFCA- and GABA-producing bacteria, which may exert anti-inflammatory effects and potentially improve mental health.

Regarding the Classification Models results, our findings demonstrate that combining stratified dietary data (nutrients, food groups, dietary indices, and dietary patterns) with microbial information better captures the behavioral differences between the two groups in both children and adolescents. This enhanced performance likely resulted from several factors: 1) reduced data complexity and redundancy through data stratification and feature selection; 2) mitigation of potential correlation among dietary variables; and 3) increased explanatory power by incorporating a broader range of relevant independent variables and potentially capturing essential interaction effects between dietary and microbial factors.

Due to the nature of Elastic-Net Penalized models, pinpointing the exact interaction types between variables can be challenging. However, examining the estimated p-values for variables within each model provides valuable insights: a general improvement in p-values was observed across selected dietary elements and combined bacterial species when compared with their performance in standalone models, suggesting a complementary effect between these two data types. This synergistic approach reveals complex relationships that might not be apparent when examining dietary components or microbial taxa in isolation. By integrating these data sources, we gain a deeper understanding of individual variability in response to diet, potentially explaining why individuals on similar diets experience different health or behavioral outcomes [95]. Therefore, the present results reveal the potential of gut microbiota data, when combined with dietary information, as a valuable tool for classification and diagnosis, an association previously recognized in literature but often overlooked in favor of single-centered dietary or taxonomical approaches [96].

This study has several limitations that must be acknowledged. First, as a cross-sectional study, it cannot establish causality between gut microbiota, diet, and behavioral problems. Second, parental reporting of behavioral problems may be biased. Third, while dividing the general population into two age groups allowed us to study diet-gut-brain interactions effectively, this approach might have limited statistical power due to the relatively small sample sizes within each group. Further studies in other geographic locations should be warranted to assess the generalizability of our main findings. The main strength of our study was the use of shotgun metagenomic sequencing, which provided a more comprehensive understanding of microbial communities than 16S rDNA sequencing used in most other studies. Furthermore, we controlled for key confounding variables influencing the gut microbiota and employed advanced statistical and mathematical modeling to investigate the complex interplay between gut microbiota, diet, and host behavior.

## Conclusions

Our findings reveal consistent associations among dietary patterns, nutrient and food group intakes, and behavioral problems in children and adolescents. Adherence to a Western diet and a deficiency in fiber-rich foods emerged as key dietary determinants that potentially impact both the gut microbiota and behavior across both age groups. While specific food items and nutrients associated with behavioral problems differed between children and adolescents; these findings align with the notion that dietary patterns may better reflect diet-microbiota interactions than individual nutrients [97]. Observed dietary differences were mirrored by alterations in the gut microbiota, characterized by decreased SCFAs-producing bacterial species and increased potentially harmful bacteria in individuals with behavioral problems. These changes may contribute to a dysfunctional gut-brain axis. Although the role of gut microbiota in mediating the effects of diet on behavior was identified in only a few cases, gut microbiota data demonstrated superior accuracy in categorizing individuals based on their behavioral phenotype compared with dietary data alone, highlighting the importance of the gut microbiota in understanding behavioral variation.

## Supplementary Information

Supplementary Material 1: Table S1. Factor loadings matrix for the three dietary patterns identified in the childhood and adolescent populations. Table S2. Associations between gut microbiota diversity and behavioral problems. Table S3. Permutation test results of Beta diversity indexes. Table S4. Differences in nutrients intake between children and adolescents with and without behavioral problems. Table S5. Differences in food group intake between children and adolescents with and without behavioral problems. Table S6. Mediation analysis of Behavioral Problems as Outcome (Y) in Adolescents. Table S7. Variable contribution summary in Elastic Net model for selected differentially abundant taxa in the children group. Table S8. Variable summary in Elastic Net comparison for full diet in the children group. Table S10. Variable contribution summary in Elastic Net model for selected differentially abundant taxa and food groups in the children group. Table S12. Variable contribution summary in Elastic Net model for selected differentially abundant taxa and dietary indexes in the children group. Table S13. Variable summary in Elastic Net comparison for selected differentially abundant taxa in the adolescent group. Table S14. Variable summary in ElasticNet comparison for full diet in the adolescents group. Table S16. Variable Summary in Elastic Net comparison for selected taxa and food groups in the adolescents group. Table S18. Variable summary in Elastic Net comparison for selected taxa and dietary patterns in the adolescent group.

## Data Availability

Sequencing data described in the manuscript will be made available once the paper is accepted for publication and other data on reasonable request to the corresponding author.

## Abbreviations

APALQ: Assessment of Physical Activity Level Questionnaire
ASD: Autism Spectrum Disorder
AUC: Area Under the Curve
z-BMI: Body Mass Index z-score
BBB: Blood–Brain Barrier
DHA: Docosahexaenoic Acid
DII: Dietary Inflammatory Index
EPA: Eicosapentaenoic Acid
FFQ: Food Frequency Questionnaire
GABA: Gamma-Aminobutyric Acid
MD: Mediterranean Diet
PCA: Principal Component Analysis
ROC: Receiving Operating Characteristic
SCFA: Short-Chain Fatty Acid
SDQ: Strengths and Difficulties Questionnaire
SENC: Spanish Society of Community Nutrition
SHEI: Spanish Healthy Eating Index
WC: Waist Circumference
