# Supplementary material for: Gut microbiota and western dietary patterns associated with behavioral problems in children and adolescents: a cross-sectional study
Source: Nutr J. 2026 May 25;25:80. doi: 10.1186/s12937-026-01335-5 (PMC13412121; doi:10.1186/s12937-026-01335-5)
Supplement: Supplementary file 1 — Supplementary Material 1: Table S1. Factor loadings matrix for the three dietary patterns identified in the childhood and adolescent populations. Table S2. Associations between gut microbiota diversity and behavioral problems. Table S3. Permutation test results of Beta diversity indexes. Table S4. Differences in nutrients intake between children and adolescents with and without behavioral problems. Table S5. Differences in food group intake between children and adolescents with and without behavioral problems. Table S6. Mediation analysis of Behavioral Problems as Outcome (Y) in Adolescents. Table S7. Variable contribution summary in Elastic Net model for selected differentially abundant taxa in the children group. Table S8. Variable summary in Elastic Net comparison for full diet in the children group. Table S10. Variable contribution summary in Elastic Net model for selected differentially abundant taxa and food groups in the children group. Table S12. Variable contribution summary in Elastic Net model for selected differentially abundant taxa and dietary indexes in the children group. Table S13. Variable summary in Elastic Net comparison for selected differentially abundant taxa in the adolescent group. Table S14. Variable summary in ElasticNet comparison for full diet in the adolescents group. Table S16. Variable Summary in Elastic Net comparison for selected taxa and food groups in the adolescents group. Table S18. Variable summary in Elastic Net comparison for selected taxa and dietary patterns in the adolescent group. [file 12937_2026_1335_MOESM1_ESM.docx]

**Additional file 1 for “Gut microbiota and Western dietary patterns associated with behavioral problems in children and adolescents: A cross-sectional study”**

**Methods**

S1 Nutritional assessment

S2 Potential confounders

S3 Mediation analysis of diet and microbiota

S4 Penalized Regression Classification Models

**Supplementary Tables**

**Table S1.** Factor loadings matrix for the three dietary patterns identified in the childhood and adolescent populations

**Table S2.**Associations between gut microbiota diversity and behavioral problems

**Table S3**. Permutation test results of Beta diversity indexes under reduced models.

**Table S4.** Differences in nutrients intake between children and adolescents with and without behavioral problems

**Table S5.** Differences in food group intake between children and adolescents with and without behavioral problems

**Table S6.** Mediation analysis results of Behavioral Problems as Outcome (Y) in Adolescents.

**Table S7.** Variable contribution summary in Elastic Net model for selected differentially abundant taxa in the children group

**Table S8.** Variable summary in Elastic Net comparison for full diet in the children group

**Table S9.** Variable contribution summary in Elastic Net model for selected differentially abundant taxa and nutrients in the children group

**Table S10.** Variable contribution summary in Elastic Net model for selected differentially abundant taxa and food groups in the children group

**Table S11.** Variable Contribution Summary in Elastic Net model for selected differentially abundant taxa and dietary patterns in the children group

**Table S12.** Variable contribution summary in Elastic Net model for selected differentially abundant taxa and dietary indexes in the children group

**Table S13.** Variable summary in Elastic Net comparison for selected differentially abundant taxa in the adolescent group

**Table S14.** Variable summary in ElasticNet comparison for full diet in the adolescents group

**Table S15.** Variable summary in ElasticNet comparison for selected taxa and nutrients in the adolescents group

**Table S16.** Variable Summary in Elastic Net comparison for selected taxa and food groups in the adolescents group

**Table S17.** Variable summary in Elastic Net comparison for selected taxa and dietary indexes in the adolescent group

**Table S18.** Variable summary in Elastic Net comparison for selected taxa and dietary patterns in the adolescent group

**Supplementary Methods**

**Nutritional Assesment**

Micro and macronutrients (g/day) were performed using consumption reported in the FFQ as well as national (CESNID and RedBEDCA) (6,7) and international (USDA) food composition tables (8). The 42 nutrients considered in the study were: Total protein, vegetal protein, animal protein, Carbohydrates, polysaccharides, intrinsic sugars, fructose, glucose, sucrose, maltose, lactose, total fiber, soluble fiber, insoluble fiber, total fats, Linolenic acid (ALA), linoleic acid (LA), Docohexaenoic acid (DHA), Eicosapentaenoic Acid (EPA), Saturated fats (SFA), Polyunsaturated fats (PUFA), Monounsaturated fats (MUFA), Cholesterol, iodine, Sodium, pottasium, calcium, magnesium, phosporus, iron, zinc, Selenium, vitamin B1, vitamin B2, vitamin B6, vitamin B12, vitamin B9, vitamin B3, vitamin C, vitamin A, vitamin D, vitamin E, flavonoids, isoflavones, antacionines, resveratrol and total polyphenols.

Food groups were evaluated using consumption reported in the FFQ. The 15 food groups considered in the study were: fresh fruit and juices, sugar beverages, legumes, nuts and olive oil, vegetables, pastries and sugars, eggs and dairy products, potatoes, processed food, whole grains, refined cereals, fish, red meat, white meat, and processed meat.

To account for variations in energy intake, we adjusted consumption (g/day) of food groups and nutrients using Willet's residual method. This method is a statistical technique used to adjust nutrient or food intakes for total energy intake, sex and age, controlling for the effect of total energy intake and socio-demographic variables involved in dietary intake (6).

*Dietary Inflammatory Index (DII)*

Calculation of DII is based on a food consumption database from eleven countries. To control the effect of energy ingestion on nutrient intake, the average intake of each parameter was adjusted by 1000 kcal. The average intake of each parameter was subtracted from the reference average of each parameter and the result was divided by the reference standard deviation of each food parameter to standardize the DII score into Z score which was converted into the corresponding percentile. The percentile value was multiplied by 2 and subtracted from 1 to obtain the centered percentile.

To achieve the specific inflammatory effect for each food parameter, the centered percentile value was multiplied by the total score of the inflammatory effect of the food parameter. Finally, the total DII was the sum of the DII for each dietary parameter, with higher scores representing more inflammatory diets (11,12)

*Adherence to the Mediterranean Diet (MD).*

The KIDMED test (Mediterranean Diet Quality Index for children and teenagers) is a tool to asses the adherence to the MD for children and adolescents. The questionnaire is based on 16-questions test which includes both positive and negative items Questions denoting a negative connotation receives a score of -1 and those with a positive aspecto +1. Therefore, the total score ranges from 0 to 12.Then, scores were classified into three levels: >8 optimal adherence to Mediterranean diet; 4–7, need improvements; ≤3 low adherence to Mediterranean diet. (7)

*Spanish Healthy Eating Index (SHEI)*

SHEI index includes nine food groups: Cereals (including pasta, rice and bread), vegetables, fruits, and dairy products, which represent daily consumption food groups; meats (including eggs, fish and meat) and legumes, which correspond to the weekly-consumption food groups; and the occasional-consumption food groups which involve processed meats; sweets (including industrial pastries, candies, cookies, mars-type chocolate, snacks, and biscuits): and sugar beverages. Finally, there is also a diet-variety variable which measures if daily or weekly recommendations are carried out correctly. These variables were divided into 5 categories that refer to the frequency of consumption: daily [1] , three or more times a week , but less than daily [2], once or twice a week [3], less than once a week [4] and never or almost never [5] (10). Higher scores indicate better diet quality and stronger adherence to the recommended dietary guidelines.

**Potential confounders**

Parents education was divided into two categories based on Spanish education: Basic education and high education. Marital status was stratified into two categories: divorced/separated/single parent or widowed and married/couple. Mode of delivery was stratified into C-section or vaginal while exclusive breastfeeding was divided into maternal or formula feeding. Bowel habits were divided into normal transit and diahrrea or constipation according to Bristol scale.

Medical history was defined as "yes" for the presence of disease or "no" for the absence of disease. Antibiotics intake were divided into “yes” as antibiotcs intake or “no” as not anbiotic intake.

Participants' stress levels were divided according to percentile classification. Participants ranked in a percentile above 70 were classified as “high stress,” while those ranked in a percentile below 70 were classified as “low stress.”

BMI z-scores for children and adolescents were calculated using AnthroPlus software, also developed by the WHO, and were categorized as follows: thinness (-3 ≤ z-score < -2), normal weight (-2 ≤ z-score ≤ +1), overweight (+1 < z-score ≤ +3), and obesity (z-score > +3). Then, z-BMI was dichotomised into normal/low weight or overweight/obesity.This classification allowed for the evaluation and comparison of the nutritional status of the participants according to international criteria (NCD Risk Factor Collaboration (NCD-RisC, 2024).

Physical activity was evaluated through the Physical Activity Level Questionnaire (APALQ) which stratified physical activity into three categories: sendentary, moderately active and active (3).

**Mediation analysis of diet and microbiota**

Mediation analysis was conducted following the Baron and Kenny framework (14). This involved three steps: examining the direct effect of the dietary element on the SDQ score; examining the effect of the dietary element on the abundance of the microbial species; and examining the combined effect of the dietary element and the microbial species on the SDQ score. If all three regressions showed statistically significant relationships, the mediation effect was further examined for causality using the R mediation package (v4.5.0) (15).

The direct effect (ADE) measures the influence of X on Y without going through M, while the Average Casual Mediation Effect (ACME) or indirect effect captures the influence of X on Y through M. Thus, the ‘mediate’ function estimates the indirect effect of Mediator and Exposure on Response and its p-value after specified bootstrapping repetitions of the model with different combinations of data (in this case, 5000 repetitions). The sum of the direct and indirect effects constitutes the total effect, providing an overall picture of how X affects Y both directly and through the mediator. Finally, a bootstrapping-based resampling method was employed to validate the identified relationships.

​
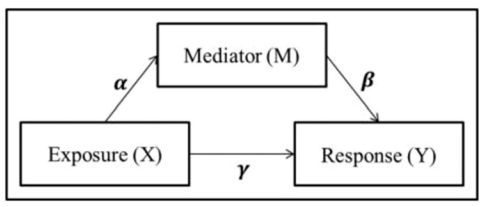
​

**Penalized regression classification models**

To enhance the performance of the Penalized Logistic Regression Models, we reduce the number of variables, first, by removing those exhibiting high levels of collinearity between microbial species and dietary elements and, second, by assessing variable importance for feature selections. For the last case, this involved incrementally adding features to a simplified logistic regression model and evaluating their contribution as classifiers using *a posteriori* Student’s t-test. For each age group (children and adolescents), seven models were developed using the following variables: Differentially Abundant Species after feature selection, All Dietary Data Combined after adjusting for correlated variables, Differentially Abundant Species and All Dietary Data Combined, Differentially Abundant Species and Nutrients, Differentially Abundant Species and Food Groups, Differentially Abundant Species and Dietary Indices, and Differentially Abundant Species and Dietary Patterns.

To address the class imbalance between Healthy and Behavioral Problem groups of adolescents, we employed a down-sampling technique using the caret package in R. This involved reducing the number of samples from the overrepresented class. To prevent overfitting, we tested multiple down-sampled datasets with varying sample combinations. Furthermore, we implemented a 70/30 train-test split to partition the data for model training and evaluation. To further mitigate overfitting, three different cross-validation methods (5-fold cross-validation, 10-fold cross-validation, and “Leave One Out” cross-validation) were tested, enabling us to choose the most optimized model.

To evaluate the performance of our models, we utilized Receiving Operator Characteristic (ROC) curve analysis, which graphically illustrates the trade-off between the model's sensitivity (correctly identifying true positives) and its specificity (correctly identifying true negatives). The Area Under the Curve (AUC) quantified the overall classificatory power of the model. A higher AUC indicates superior model performance in distinguishing subjects with high Global SDQ scores from those with low scores.

**Table S1.** Factor loadings matrix for the three dietary patterns identified in the childhood and adolescent populations

| **Food group** | **Childhood dietary patterns** | | | | **Adolescence dietary patterns** | | |
| --- | --- | --- | --- | --- | --- | --- | --- |
|  | Plant-based diet | | Western  Diet | Meat and cereals-based diet | Plant-based diet | Western  diet | Meat-based diet |
| Sugar Beverage (g) | | 0.1 | **0.8** | 0.1 | -0.1 | **0.8** | 0.1 |
| Fresh fruit(g) | | **0.7** | -0.1 | 0.1 | **0.5** | -0.1 | -0.2 |
| Legumes(g) | | **0.7** | 0.1 | -0.1 | **0.8** | 0.1 | -0.1 |
| Nuts and oil olive(g) | | 0.2 | 0.2 | 0.0 | 0.2 | -0.1 | -0.3 |
| Vegetables(g) | | **0.8** | 0.0 | 0.1 | **0.8** | -0.1 | 0.1 |
| Processed food(g) | | -0.1 | **0.5** | 0.2 | -0.1 | **0.7** | 0.3 |
| Cheesee(g) | | 0.0 | -0.1 | 0.1 | 0.0 | 0.3 | -0.1 |
| Pastries and sugars(g) | | -0.2 | **0.7** | 0.2 | 0.2 | -0.1 | 0.1 |
| Yogurt and milk(g) | | 0.1 | 0.2 | 0.0 | 0.0 | -0.1 | 0.0 |
| Potatoes(g) | | 0.2 | 0.1 | 0.0 | 0.0 | 0.3 | 0.2 |
| Whole grain(g) | | **0.5** | 0.0 | -0.2 | **0.4** | -0.3 | 0.0 |
| Refined cereals(g) | | 0.0 | 0.3 | **0.7** | 0.0 | 0.0 | 0.1 |
| Fish(g) | | 0.2 | **0.7** | 0.0 | 0.2 | -0.3 | 0.2 |
| Red meat(g) | | 0.0 | 0.3 | **0.7** | 0.0 | 0.1 | **0.9** |
| Whitemeat(g) | | 0.0 | 0.1 | 0.3 | 0.0 | -0.1 | **0.5** |
| Processed meat(g) | | -0.2 | **0.4** | 0.3 | -0.2 | **0.4** | **0.6** |

The values presented are the factor loadings extracted by Principal Component Analysis (PCA) with varimax rotation. Loadings equal or greater than 0.4 in bold. Components are labeled based on the variables with the highest loadings on each factor

| **Table S2** Associations between gut microbiota diversity and behavioral problems | | | |
| --- | --- | --- | --- |
| **Childhood** | **β (95% CI)** | **p** | **q*** |
| Chao1 | 0.002(-0.005, 0.008) | 0.55 | 0.98 |
| Shannon | 0.68 ( -0.07, 1.463) | 0.08 | 0.15 |
| Inv. Simpson | 0.57 (0.008, 0.106) | **0.02** | **0.03** |
| **Adolescence** | **β (95% CI)** | **p** | **q*** |
| Chao1 | 0.006 (0.004, 0.015) | 0.23 | 0.12 |
| Shannon | 0.97 (-0.13, 2.07) | 0.08 | 0.06 |
| Inv. Simpson | 0.77 (0.008, 0.146) | **0.03** | **0.02** |

β (95% CI) Coefficient of the independent variable, representing an odds ratio. p: Model not adjusted for covariables (P<0.05). q: FDR-adjusted by age, sex, z-BMI, parent education, bowel movements, APALQ, stress level and medication and antibiotic intake. Significant differences in bold (p<0.05)

| Table S3: Permutation test results of Beta diversity indexes | | | | | | |
| --- | --- | --- | --- | --- | --- | --- |
| Children |  | Df | SumOfSqs | R2 | F | p |
| **Bray-Curtis** | Model | 1 | 0.229 | 0.00682 | 1.3738 | 0.147 |
|  | Residual | 200 | 33.286 | 0.99318 |  |  |
|  | Total | 201 | 33.515 | 1.00000 |  |  |
| **Jaccard** | Model | 1 | 0.350 | 0.00669 | 1.3738 | 0.096 |
|  | Residual | 200 | 52.014 | 0.99331 |  |  |
|  | Total | 201 | 52.364 | 1.00000 |  |  |
|  |  |  |  |  |  |  |
| Adolescents |  | Df | SumOfSqs | R2 | F | p |
| **Bray-Curtis** | Model | 1 | 0.196 | 0.00943 | 1.2474 | 0.218 |
|  | Residual | 131 | 20.580 | 0.99057 |  |  |
|  | Total | 132 | 20.776 | 1.00000 |  |  |
| **Jaccard** | Model | 1 | 0.310 | 0.00934 | 1.2355 | 0.186 |
|  | Residual | 131 | 32.898 | 0.99066 |  |  |
|  | Total | 132 | 33.209 | 1.00000 |  |  |

Df: degrees of freedom. SumOfSqs: Sum of Squares.

**Table S4.** Differences in nutrients intake between children and adolescents with and without behavioral problems

| **Nutrient intake** **(g/day)** | **Children (5-10 years)**  **N=202 (61%)** | | | | | **Adolescents (11-17 years)**  **N=133 (39%)** | | | | |
| --- | --- | --- | --- | --- | --- | --- | --- | --- | --- | --- |
|  | **Healthy** | | **Behavioral problems** | | **p** | **Healthy** | | **Behavioral problems** | | **p** |
|  | **M** | **SD** | **M** | **SD** |  | **M** | **SD** | **M** | **SD** |  |
| Total protein (g) | 85.0 | 2.0 | 85.9 | 2.5 | 0.40 | 114.8 | 3.6 | 115.5 | 5.0 | 0.88 |
| Vegetal protein (g) | 33.0 | 1.6 | 30.5 | 1.7 | 0.07 | 37.7 | 1.9 | 35.9 | 2.3 | 0.72 |
| Animal protein (g) | 57.7 | 2.5 | 59.9 | 2.5 | 0.20 | 77.1 | 4.2 | 79.6 | 5.2 | 0.58 |
| Carbohydrates(g) | 220.0 | 5.6 | 210.3 | 4.8 | 0.17 | 245.5 | 7.6 | 246.4 | 9.5 | 0.84 |
| Polysacchares (g) | 130.4 | 5.3 | 126.2 | 5.6 | 0.31 | 147.3 | 7.2 | 158.4 | 10.7 | 0.54 |
| Intrinsic sugars (g) | 100.6 | 4.6 | 98.3 | 5.1 | 0.34 | 111.0 | 5.4 | 102.9 | 6.8 | 0.44 |
| Fructose (g) | 27.3 | 2.3 | 22.4 | 2.4 | **0.06** | 27.5 | 2.4 | 22.5 | 3.1 | 0.37 |
| Glucose (g) | 19.0 | 1.6 | 15.4 | 1.6 | **0.09** | 20.2 | 1.9 | 17.3 | 2.6 | 0.63 |
| Sucrose (g) | 3.3 | 0.2 | 3.0 | 0.3 | 0.13 | 4.3 | 0.4 | 4.6 | 0.6 | 0.87 |
| Maltose (g) | 26.8 | 1.6 | 25.3 | 1.6 | 0.34 | 28.7 | 2.2 | 24.9 | 2.5 | 0.59 |
| Lactose (g) | 32.1 | 2.8 | 37.2 | 3.4 | 0.38 | 38.0 | 3.7 | 37.6 | 5.0 | 0.29 |
| Total fiber (g) | 27.5 | 1.9 | 23.3 | 1.7 | **0.04** | 31.5 | 2.0 | 25.9 | 2.5 | 0.10 |
| Soluble fiber(g) | 7.0 | 0.4 | 5.9 | 0.5 | **0.01** | 8.1 | 0.5 | 6.9 | 0.6 | 0.13 |
| Insoluble fiber (g) | 19.9 | 1.5 | 16.6 | 1.4 | **0.04** | 22.1 | 1.5 | 18.1 | 1.9 | 0.13 |
| Total fats (g) | 68.8 | 1.7 | 71.8 | 1.7 | 0.16 | 89.5 | 2.7 | 84.3 | 3.7 | 0.26 |
| Linolenic acid (ALA) (g) | 1.4 | 0.2 | 1.2 | 0.1 | 0.93 | 2.2 | 0.2 | 2.0 | 0.3 | 0.75 |
| linoleic acid (LA) (g) | 4.8 | 0.3 | 4.5 | 0.2 | 0.79 | 5.8 | 0.4 | 5.3 | 0.4 | 0.46 |
| Docohexaenoic acid (DHA) (mg) | 391.8 | 35.3 | 327.4 | 37.4 | 0.21 | 460.7 | 55.9 | 688.0 | 91.4 | **0.03** |
| Eicosapentaenoic Acid (EPA) (mg) | 214.2 | 20.9 | 184.1 | 21.4 | 0.36 | 284.9 | 35.8 | 407.6 | 55.7 | 0.06 |
| Saturated fats (SFA) (g) | 22.5 | 0.7 | 24.3 | 0.9 | 0.22 | 29.5 | 1.2 | 27.6 | 1.6 | 0.40 |
| Polyunsaturated fats (PUFA) (g) | 10.1 | 0.3 | 10.8 | 0.5 | 0.28 | 13.3 | 0.6 | 12.9 | 0.9 | 0.37 |
| Monounsaturated fats (MUFA) (g) | 30.3 | 0.8 | 30.0 | 0.8 | 1.00 | 38.9 | 1.1 | 36.8 | 1.7 | 0.25 |
| Cholesterol (mg) | 310.2 | 14.1 | 340.0 | 16.2 | 0.09 | 352.7 | 15.9 | 352.6 | 20.7 | 0.92 |
| Iodine (µg) | 118.9 | 5.5 | 115.5 | 6.0 | 0.47 | 134.9 | 6.2 | 137.6 | 10.5 | 0.99 |
| Sodium (mg) | 1908.0 | 68.0 | 2003.5 | 78.0 | 0.32 | 2758.2 | 111.4 | 2847.6 | 170.6 | 0.84 |
| Potassium (mg) | 3332.9 | 118.7 | 3149.9 | 128.2 | 0.22 | 4091.9 | 163.5 | 3800.3 | 199.2 | 0.29 |
| Calcium (mg) | 914.1 | 42.4 | 932.6 | 55.0 | 0.93 | 1112.9 | 68.4 | 1043.2 | 88.0 | 0.63 |
| Magnesium (mg) | 341.9 | 10.3 | 327.3 | 11.4 | 0.32 | 403.4 | 15.2 | 383.4 | 24.1 | 0.25 |
| Phosphorus (mg) | 1235.6 | 28.4 | 1241.4 | 41.1 | 0.77 | 1481.4 | 43.1 | 1516.8 | 55.7 | 0.57 |
| Iron (mg) | 13.4 | 0.5 | 12.3 | 0.5 | 0.16 | 16.6 | 0.7 | 16.0 | 1.1 | 0.45 |
| Zinc (mg) | 9.7 | 0.2 | 9.8 | 0.3 | 0.74 | 12.5 | 0.4 | 12.1 | 0.5 | 0.57 |
| Selenium (µg) | 97.8 | 4.4 | 92.0 | 4.6 | 0.54 | 113.1 | 5.2 | 119.8 | 8.4 | 0.75 |
| Vitamin B1 (mg) | 1.6 | 0.1 | 1.5 | 0.1 | 0.15 | 2.0 | 0.1 | 2.0 | 0.1 | 0.86 |
| Vitamin B2 (mg) | 1.9 | 0.1 | 1.9 | 0.1 | 0.71 | 2.2 | 0.1 | 2.2 | 0.1 | 0.99 |
| Vitamin B6 (mg) | 2.4 | 0.1 | 2.2 | 0.1 | 0.14 | 3.0 | 0.1 | 3.0 | 0.2 | 0.71 |
| Vitamin B12 (µg) | 7.0 | 0.3 | 7.1 | 0.3 | 0.70 | 8.3 | 0.4 | 8.5 | 0.6 | 0.75 |
| Vitamin B9 (µg) | 351.8 | 23.5 | 282.8 | 17.6 | **0.02** | 377.1 | 23.4 | 337.9 | 33.4 | 0.33 |
| Vitamin B3 (mg) | 23.4 | 0.8 | 23.1 | 0.9 | 0.58 | 31.0 | 1.2 | 33.5 | 1.7 | 0.27 |
| Vitamin C (mg) | 191.0 | 16.8 | 160.2 | 15.5 | 0.25 | 255.8 | 23.0 | 199.0 | 31.3 | 0.15 |
| Vitamin A (µg) | 1335.2 | 123.3 | 1344.0 | 230.8 | 0.76 | 1551.2 | 136.9 | 1205.3 | 173.1 | 0.13 |
| Vitamin D (µg) | 4.2 | 0.4 | 4.8 | 0.6 | 0.79 | 6.0 | 0.6 | 6.5 | 0.8 | 0.60 |
| Vitamin E (mg) | 11.1 | 0.5 | 9.8 | 0.5 | 0.05 | 13.0 | 0.6 | 11.0 | 0.9 | 0.06 |
| Flavonoids (mg) | 839.6 | 96.1 | 985.2 | 106.2 | 0.36 | 1259.5 | 166.6 | 1309.6 | 256.7 | 0.96 |
| Isoflavones (mg) | 52.2 | 13.9 | 72.6 | 17.8 | 0.35 | 204.2 | 61.6 | 287.0 | 102.2 | 0.49 |
| Antacionines (mg) | 130.6 | 20.8 | 112.9 | 21.1 | 0.73 | 127.4 | 19.4 | 76.7 | 21.3 | 0.10 |
| Resveratrol (mg) | 0.3 | 0.0 | 0.3 | 0.1 | 0.41 | 0.4 | 0.1 | 0.2 | 0.1 | 0.15 |
| Total polyphenols (mg) | 2165.0 | 211.7 | 2088.8 | 214.2 | 0.86 | 2556.5 | 194.2 | 2466.5 | 279.3 | 0.79 |

* Mean (SD). Mann-Whitney U, Kruskal-Wallis and Student’s t-tests were used for statistical analysis. Significant differences in bold (p<0.05)

**Table S5**. Differences in food group intake between children and adolescents with and without behavioral problems

|  | **Children (5-10 years) N= 202 (61%)** | | | | | **Adolescents (11-17 years) N=133 (39%)** | | | | |
| --- | --- | --- | --- | --- | --- | --- | --- | --- | --- | --- |
|  | Healthy | | Behavioral problems | | p | Healthy | | Behavioral problems | | p |
|  | M | SD | M | SD |  | M | SD | M | SD |  |
| Sugar Beverages (g) | 166.2 | 35.6 | 167.5 | 25.2 | 0.89 | 186.7 | 33.0 | 182.8 | 42.1 | 0.71 |
| Fresh fruit and juices (g) | 428.6 | 36.0 | 334.8 | 32.8 | **0.04** | 502.6 | 51.3 | 435.5 | 70.3 | 0.48 |
| Legumes (g) | 63.3 | 8.8 | 55.1 | 8.9 | 0.16 | 62.9 | 9.4 | 45.9 | 12.2 | **0.04** |
| Nuts and oil olive (g) | 11.8 | 2.1 | 10.0 | 2.2 | 0.20 | 11.2 | 2.3 | 9.3 | 3.2 | **0.03** |
| Vegetables (g) | 521.0 | 58.4 | 469.2 | 58.4 | 0.70 | 793.4 | 75.5 | 457.3 | 79.4 | **0.04** |
| Processed food (g) | 42.1 | 3.5 | 57.1 | 5.0 | 0.11 | 63.0 | 6.7 | 75.4 | 11.6 | 0.96 |
| Eggs (g) | 27.9 | 2.1 | 27.2 | 2.5 | 0.32 | 27.8 | 3.1 | 32.3 | 4.1 | 0.26 |
| Cheese (g) | 24.7 | 3.6 | 33.0 | 4.9 | 0.31 | 40.3 | 5.3 | 39.7 | 7.6 | 0.90 |
| Pastries and sugars (g) | 103.0 | 11.2 | 127.9 | 14.1 | 0.21 | 80.1 | 11.5 | 79.7 | 16.2 | 0.41 |
| Yogurt and milk (g) | 460.4 | 45.7 | 511.7 | 60.1 | 0.80 | 477.7 | 54.2 | 440.9 | 65.1 | 0.66 |
| Potatoes (g) | 71.5 | 7.4 | 87.1 | 9.1 | 0.20 | 97.8 | 12.6 | 116.8 | 19.1 | 0.16 |
| Whole grain (g) | 30.4 | 5.5 | 28.5 | 6.4 | 0.50 | 54.5 | 12.0 | 47.3 | 12.5 | 0.61 |
| Refined cereals (g) | 121.8 | 10.4 | 110.2 | 11.8 | 0.37 | 154.8 | 15.5 | 159.8 | 24.1 | 0.98 |
| Fish (g) | 72.1 | 5.7 | 64.2 | 6.7 | 0.20 | 97.3 | 12.3 | 135.6 | 17.9 | **0.02** |
| Red meat (g) | 58.7 | 6.8 | 64.2 | 7.8 | 0.50 | 103.7 | 14.1 | 106.9 | 17.3 | 0.57 |
| White meat (g) | 69.3 | 6.0 | 72.4 | 6.8 | 0.38 | 105.5 | 9.1 | 91.2 | 10.0 | 0.78 |
| Processed meat (g) | 19.5 | 1.8 | 24.6 | 2.3 | 0.23 | 49.9 | 9.0 | 48.9 | 13.5 | 0.75 |

* Data are expressed as means (SD); Mann-Whitney U, Kruskal-Wallis and Student’s t-tests were used for statistical analysis. Significant differences are in bold (p<0.05).

| Table S6: Mediation analysis results of Behavioral Problems as Outcome (Y) in Adolescents. | | | | | |
| --- | --- | --- | --- | --- | --- |
| *Anaerostipes rhamnosivorans (M) → Fish and Seafood (X)* | | | | | |
|  | Estimate | | 95% CI Lower | 95% CI Upper | p |
| ACME | 0.097 | | 0.028 | 0.18 | **0.03** |
| ADE | 0.0236 | | 0.002 | 0.05 | **0.008** |
| Total Effect | 0.12 | | 0.04 | 0.03 | **2E-16** |
| *Lautropia mirabilis (M) →* Western Dietary Pattern (X) | | | | | |
|  | Estimate | 95% CI Lower | | 95% CI Upper | p |
| ACME | 0.004 | -0.006 | | 0.09 | **0.02** |
| ADE | 0.072 | -0.008 | | 0.01 | 0.078 |
| Total Effect | 0.116 | 0.025 | | 0.26 | **0.018** |

ACME: Average Causal Mediated Effect. ADE: Average Direct Effect. CI: Coefficient Interval. M: Mediator variable. X: Exposure variable. Y: Response variable. Significant estimates in bold (p<0.05).

| **Table S7.** Variable contribution summary in Elastic Net model for selected differentially abundant taxa in the children group | | | | | |
| --- | --- | --- | --- | --- | --- |
| Variable | β-coefficients | Standard Error | **Exp(β)** | CI (95%) | *P value* |
| *(Intercept)* | -0.57 | 0.32 | 5.66E-01 | [0.299, 1.05] | 0.07 |
| *Campylobacter coli* | 0.49 | 0.17 | 1.64E+00 | [1.2, 2.29] | 0.00 |
| *Bifidobacterium pullorum* | 0.36 | 0.17 | 1.43E+00 | [1.03, 2.02] | 0.04 |
| *Latilactobacillus sakei* | 0.28 | 0.16 | 1.33E+00 | [0.973, 1.82] | 0.08 |
| *Intestinimonas butyriciproducens* | -0.16 | 0.16 | 8.55E-01 | [0.618, 1.18] | 0.34 |
| *Butyrivibrio fibrisolvens* | -0.48 | 0.17 | 6.16E-01 | [0.435, 0.853] | 0.00 |
| *Age* | 0.37 | 0.20 | 1.45E+00 | [0.98, 2.16] | 0.07 |
| *Sex* | -0.48 | 0.32 | 6.18E-01 | [0.329, 1.15] | 0.13 |
| *Antibiotics* | 1.01 | 0.50 | 2.75E+00 | [1.06, 7.53] | 0.04 |

Abbreviations: Exp (β) = Exponential of the coefficient β, representing an odds ratio; CI: Confidence intervals

| Table S8. Variable summary in Elastic Net comparison for full diet in the children group | | | | | |
| --- | --- | --- | --- | --- | --- |
| Variable | β-coefficients | Standard Error | **Exp(β)** | CI (95%) | *P value* |
| *Total protein (g)* | 0.04 | 0.04 | 1.00 | [0.97, 1.1] | 0.26 |
| *Carbohydrates (g)* | -0.01 | 0.01 | 0.99 | [0.96, 1] | 0.40 |
| *Polysacchares (g)* | 0.00 | 0.02 | 1.00 | [0.97, 1] | 0.88 |
| *Intrinsic sugars (g)* | 0.02 | 0.02 | 1.00 | [0.99, 1.1] | 0.24 |
| *Fructose (g)* | -0.03 | 0.02 | 0.97 | [0.94, 1] | 0.09 |
| *Glucose (g)* | 0.01 | 0.04 | 1.00 | [0.93, 1.1] | 0.87 |
| *Sucrose (g)* | -0.18 | 0.13 | 0.84 | [0.65, 1.1] | 0.16 |
| *Maltose (g)* | -0.02 | 0.02 | 0.98 | [0.95, 1] | 0.39 |
| *Lactose (g)* | 0.11 | 0.12 | 1.10 | [0.88, 1.4] | 0.38 |
| *Total fiber (g)* | 0.03 | 0.27 | 1.00 | [0.61, 1.8] | 0.90 |
| *Soluble fiber(g)* | -0.00 | 0.12 | 1.00 | [0.79, 1.3] | 0.98 |
| *Total fats (g)* | -0.00 | 0.00 | 1.00 | [0.99, 1] | 0.22 |
| *Linolenic acid (ALA) (g)* | 0.01 | 0.01 | 1.00 | [0.99, 1] | 0.38 |
| *linoleic acid (LA) (g)* | -0.04 | 0.07 | 0.96 | [0.83, 1.1] | 0.58 |
| *Docohexaenoic acid (DHA) (mg)* | -0.01 | 0.07 | 0.98 | [0.86, 1.1] | 0.82 |
| *Eicosapentaenoic acid (EPA) (mg)* | -0.01 | 0.01 | 0.99 | [0.98, 1] | 0.22 |
| *Saturated fats (SFA) (g)* | 0.00 | 0.00 | 1.00 | [1, 1] | 0.65 |
| *Polyunsaturated fats (PUFA) (g)* | -0.00 | 0.00 | 1.00 | [0.99, 1] | 0.55 |
| *Monounsaturated fats (MUFA) (g)* | 0.00 | 0.00 | 1.00 | [1, 1] | 0.99 |
| *Cholesterol (mg)* | -0.05 | 0.12 | 0.95 | [0.76, 1.2] | 0.65 |
| *Iodine (µg)* | 0.01 | 0.01 | 1.00 | [0.98, 1] | 0.61 |
| *Sodium (mg)* | -0.31 | 0.57 | 0.73 | [0.24, 2.2] | 0.58 |
| *Calcium (mg)* | 0.20 | 0.89 | 1.20 | [0.22, 7] | 0.82 |
| *Magnesium (mg)* | -0.29 | 0.75 | 0.75 | [0.17, 3.3] | 0.70 |
| *Phosphorus (mg)* | -0.01 | 0.16 | 0.99 | [0.72, 1.4] | 0.95 |
| *Iron (mg)* | 0.06 | 0.07 | 1.10 | [0.93, 1.2] | 0.35 |
| *Selenium (µg)* | -0.14 | 0.13 | 0.87 | [0.68, 1.1] | 0.29 |
| *Vitamin B2 (mg)* | 0.00 | 0.00 | 1.00 | [1, 1] | 0.44 |
| *Vitamin B12 (µg)* | 0.00 | 0.00 | 1.00 | [1, 1] | 0.49 |
| *Vitamin B9 (µg)* | -0.01 | 0.00 | 0.99 | [0.99, 1] | 0.01 |
| *Vitamin B3 (mg)* | 0.11 | 0.06 | 1.10 | [0.99, 1.3] | 0.06 |
| *Vitamin C (mg)* | 0.00 | 0.00 | 1.00 | [1, 1] | 0.23 |
| *Vitamin A (µg)* | 0.00 | 0.00 | 1.00 | [1, 1] | 0.17 |
| *Vitamin D (µg)* | 0.31 | 0.65 | 1.40 | [0.38, 4.9] | 0.63 |
| *Vitamin E (mg)* | -0.00 | 0.00 | 1.00 | [1, 1] | 0.42 |
| *Flavonoids (mg)* | -0.05 | 0.11 | 0.95 | [0.77, 1.2] | 0.63 |
| *Isoflavones (mg)* | -0.05 | 0.03 | 0.95 | [0.9, 1] | 0.12 |
| *Antacionines (mg)* | -0.01 | 0.05 | 0.99 | [0.89, 1.1] | 0.88 |
| *Resveratrol (mg)* | -0.00 | 0.08 | 1.00 | [0.85, 1.2] | 0.98 |
| *Total polyphenols (mg)* | 0.03 | 0.06 | 1.00 | [0.92, 1.2] | 0.59 |
| *Fresh fruit(g)* | 0.03 | 0.04 | 1.00 | [0.94, 1.1] | 0.56 |
| *Sugar Beverage (g)* | -0.09 | 0.18 | 0.91 | [0.65, 1.3] | 0.61 |
| *Legumes(g)* | 0.04 | 0.06 | 1.00 | [0.93, 1.2] | 0.49 |
| *Nuts and oil olive(g)* | -0.10 | 0.20 | 0.91 | [0.61, 1.3] | 0.64 |
| *Vegetables(g)* | 0.01 | 0.04 | 1.00 | [0.94, 1.1] | 0.76 |
| *Eggs (g)* | 0.02 | 0.02 | 1.00 | [0.98, 1.1] | 0.32 |
| *Cheese (g)* | 0.01 | 0.04 | 1.00 | [0.94, 1.1] | 0.83 |
| *Pastries and sugars(g)* | 0.02 | 0.03 | 1.00 | [0.97, 1.1] | 0.37 |
| *Yogurt and milk(g)* | 0.03 | 0.03 | 1.00 | [0.98, 1.1] | 0.24 |
| *Potatoes(g)* | -0.01 | 0.04 | 0.99 | [0.92, 1.1] | 0.82 |
| *Processed food(g)* | 0.01 | 0.04 | 1.00 | [0.93, 1.1] | 0.87 |
| *Whole grain(g)* | -0.03 | 0.07 | 0.97 | [0.84, 1.1] | 0.68 |
| *Refined cereals(g)* | -0.02 | 0.03 | 0.98 | [0.93, 1] | 0.42 |
| *Fish(g)* | 0.06 | 0.05 | 1.10 | [0.96, 1.2] | 0.28 |
| *Red meat(g)* | 0.69 | 2.30 | 2.00 | [0.023, 170] | 0.76 |
| *Whitemeat(g)* | 0.21 | 0.48 | 1.20 | [0.48, 3.1] | 0.67 |
| *Processed meat(g)* | -0.20 | 0.31 | 0.82 | [0.44, 1.5] | 0.53 |
| *Plant-based diet* | -0.10 | 0.20 | 0.90 | [0.61, 1.3] | 0.61 |
| *Western diet* | -0.00 | 0.04 | 1.00 | [0.92, 1.1] | 0.96 |
| *Meat and cereals diet* | -0.12 | 0.57 | 0.88 | [0.29, 2.7] | 0.83 |
| *SHEI* | 0.83 | 0.55 | 2.30 | [0.77, 6.7] | 0.13 |
| *SHEIQ* | 0.00 | 0.25 | 1.00 | [0.61, 1.6] | 1.00 |
| *MDQ* | 1.90 | 0.64 | 6.90 | [2, 24] | 0.00 |
| *DII* | 0.16 | 0.23 | 1.20 | [0.74, 1.8] | 0.50 |
| *DIIQ* | -0.13 | 0.27 | 0.88 | [0.52, 1.5] | 0.63 |

| **Table S9.** Variable contribution summary in Elastic Net model for selected differentially abundant taxa and nutrients in the children group | | | | | |  |
| --- | --- | --- | --- | --- | --- | --- |
| Variable | β-coefficients | Std. Error | **Exp(β)** | CI (95%) | Pr(>\|z\|) | |
| *Campylobacter coli* | 0.48 | 0.17 | 1.61E+00 | [1.17, 2.26] | 0.00 | |
| *Bifidobacterium pullorum* | 0.39 | 0.17 | 1.47E+00 | [1.06, 2.09] | 0.02 | |
| *Butyrivibrio fibrisolvens* | -0.46 | 0.17 | 6.30E-01 | [0.443, 0.878] | 0.01 | |
| *Antibiotics* | 1.25 | 0.53 | 3.50E+00 | [1.28, 10.2] | 0.02 | |
| *Vitamin B9 (mg)* | -0.38 | 0.18 | 6.88E-01 | [0.468, 0.967] | 0.04 | |

Abbreviations: Exp (β) = Exponential of the coefficient β, representing an odds ratio; CI: Confidence intervals

| **Table S10.** Variable contribution summary in Elastic Net model for selected differentially abundant taxa and food groups in the children group | | | | | |
| --- | --- | --- | --- | --- | --- |
| Variable | β-coefficients | Standard Error | **Exp(β)** | CI (95%) | *P value* |
| *Campylobacter coli* | 0.49 | 0.17 | 1.60 | [1.2, 2.3] | 0.00 |
| *Bifidobacterium pullorum* | 0.37 | 0.17 | 1.50 | [1, 2] | 0.03 |
| *Latilactobacillus sakei* | 0.26 | 0.16 | 1.30 | [0.95, 1.8] | 0.10 |
| *Intestinimonas butyriciproducens* | -0.12 | 0.17 | 0.89 | [0.64, 1.2] | 0.47 |
| *Butyrivibrio fibrisolvens* | -0.43 | 0.17 | 0.65 | [0.47, 0.91] | 0.01 |
| *Age* | 0.13 | 0.21 | 1.10 | [0.75, 1.7] | 0.53 |
| *Sex* | -0.46 | 0.32 | 0.63 | [0.34, 1.2] | 0.15 |
| *Antibiotics* | 1.20 | 0.50 | 3.40 | [1.3, 9] | 0.01 |
| *z-BMI* | -0.14 | 0.40 | 0.87 | [0.39, 1.9] | 0.73 |
| *Pastries (g)* | 0.26 | 0.17 | 1.30 | [0.93, 1.8] | 0.12 |
| *Potatoes (g)* | 0.27 | 0.17 | 1.30 | [0.94, 1.8] | 0.12 |
| *Processed food (g)* | 0.30 | 0.18 | 1.30 | [0.95, 1.9] | 0.09 |
| *Whitemeat (g)* | 0.23 | 0.17 | 1.30 | [0.91, 1.7] | 0.17 |

| Abbreviations: Exp (β) = Exponential of the coefficient β, representing an odds ratio; CI: Confidence intervals | | | | | |
| --- | --- | --- | --- | --- | --- |
| **Table S11.** Variable Contribution Summary in Elastic Net model for selected differentially abundant taxa and dietary patterns in the children group | | | | | |
| Variable | β-coefficients | Standard Error | **Exp(β)** | CI(95%) | *P value* |
| *Campylobacter coli* | 0.48 | 0.16 | 1.60 | [1.2, 2.2] | 0.00 |
| *Bifidobacterium pullorum* | 0.34 | 0.16 | 1.40 | [1, 1.9] | 0.04 |
| *Latilactobacillus sakei* | 0.29 | 0.16 | 1.30 | [0.98, 1.8] | 0.06 |
| *Intestinimonas butyriciproducens* | -0.15 | 0.16 | 0.86 | [0.63, 1.2] | 0.36 |
| *Butyrivibrio fibrisolvens* | -0.44 | 0.17 | 0.64 | [0.46, 0.89] | 0.01 |
| *Age* | 0.30 | 0.20 | 1.40 | [0.91, 2] | 0.13 |
| *Sex* | -0.42 | 0.31 | 0.66 | [0.36, 1.2] | 0.18 |
| *Antibiotics* | 1.10 | 0.49 | 3.20 | [1.2, 8.2] | 0.02 |
| *z-BMI* | -0.06 | 0.38 | 0.94 | [0.45, 2] | 0.88 |

Abbreviations: Exp (β) = Exponential of the coefficient β, representing an odds ratio; CI: Confidence intervals

| \| **Table S12.** Variable contribution summary in Elastic Net model for selected differentially abundant taxa and dietary indexes in the children group \| \| \| \| \| \| \| --- \| --- \| --- \| --- \| --- \| --- \| \| Variable \| β-coefficients \| Standard Error \| **Exp(β)** \| CI (95%) \| *P value* \| \| *Campylobacter coli* \| 0.51 \| 0.16 \| 1.70 \| [1.2, 2.3] \| 0.00 \| \| *Bifidobacterium pullorum* \| 0.35 \| 0.17 \| 1.40 \| [1, 2] \| 0.04 \| \| *Latilactobacillus sakei* \| 0.29 \| 0.16 \| 1.30 \| [0.98, 1.8] \| 0.06 \| \| *Intestinimonas butyriciproducens* \| -0.11 \| 0.16 \| 0.89 \| [0.65, 1.2] \| 0.49 \| \| *Butyrivibrio fibrisolvens* \| -0.44 \| 0.17 \| 0.65 \| [0.46, 0.9] \| 0.01 \| \| *Age* \| 0.33 \| 0.20 \| 1.40 \| [0.94, 2.1] \| 0.10 \| \| *Sex* \| -0.36 \| 0.32 \| 0.70 \| [0.37, 1.3] \| 0.26 \| \| *Antibiotics* \| 1.10 \| 0.49 \| 2.90 \| [1.1, 7.5] \| 0.03 \| \| *SHEI* \| -0.23 \| 0.16 \| 0.79 \| [0.58, 1.1] \| 0.16 \| |
| --- | --- | --- | --- | --- | --- | --- | --- | --- | --- | --- | --- | --- | --- | --- | --- | --- | --- | --- | --- | --- | --- | --- | --- | --- | --- | --- | --- | --- | --- | --- | --- | --- | --- | --- | --- | --- | --- | --- | --- | --- | --- | --- | --- | --- | --- | --- | --- | --- | --- | --- | --- | --- | --- | --- | --- | --- | --- | --- | --- | --- | --- | --- | --- | --- | --- | --- |

Abbreviations: Exp (β) = Exponential of the coefficient β, representing an odds ratio; CI: Confidence intervals

| **Table S13.** Variable summary in Elastic Net comparison for selected differentially abundant taxa in the adolescent group | | | | | | |
| --- | --- | --- | --- | --- | --- | --- |
| Variable | β-coefficients | Standard Error | **Exp(β)** | CI (95%) | *P value* |  |
| *Butyricimonas virosa* | -0.52 | 0.22 | 0.59 | [0.39, 0.91] | 0.02 |  |
| *Parolsenella catena* | -0.67 | 0.26 | 0.51 | [0.3, 0.86] | 0.01 |  |
| *Ruminococcus champanellensis* | 0.59 | 0.22 | 1.80 | [1.2, 2.8] | 0.01 |  |
| *Anaerostipes rhamnosivorans* | 0.54 | 0.22 | 1.70 | [1.1, 2.6] | 0.02 |  |
| *Age* | -0.52 | 0.36 | 0.59 | [0.29, 1.2] | 0.15 |  |
| *Sex* | -0.37 | 0.42 | 0.69 | [0.3, 1.6] | 0.39 |  |
| *z-BMI* | 0.60 | 0.35 | 1.80 | [0.93, 3.6] | 0.08 |  |

Abbreviations: Exp (β) = Exponential of the coefficient β, representing an odds ratio; CI: Confidence intervals

| **Table S14.** Variable summary in ElasticNet comparison for full diet in the adolescents group | | | | | | |
| --- | --- | --- | --- | --- | --- | --- |
| Variable | β-coefficients | Standard Error | **Exp(β)** | CI (95%) | P value |  |
| *Total protein (g)* | -0.03 | 0.03 | 0.97 | [0.91, 1] | 0.28 |  |
| *Carbohydrates(g)* | -0.01 | 0.02 | 0.99 | [0.95, 1] | 0.71 |  |
| *Polysacchares (g)* | 0.01 | 0.02 | 1.00 | [0.96, 1.1] | 0.76 |  |
| *Intrinsic sugars (g)* | 0.01 | 0.03 | 1.00 | [0.95, 1.1] | 0.76 |  |
| *Fructose (g)* | -0.02 | 0.03 | 0.98 | [0.92, 1] | 0.41 |  |
| *Glucose (g)* | 0.00 | 0.05 | 1.00 | [0.9, 1.1] | 0.96 |  |
| *Sucrose (g)* | 0.12 | 0.19 | 1.10 | [0.78, 1.6] | 0.53 |  |
| *Maltose (g)* | -0.04 | 0.03 | 0.96 | [0.91, 1] | 0.20 |  |
| *Lactose (g)* | -0.01 | 0.03 | 0.99 | [0.94, 1] | 0.73 |  |
| *Total fiber (g)* | 0.06 | 0.10 | 1.10 | [0.88, 1.3] | 0.53 |  |
| *Soluble fiber(g)* | -0.36 | 0.28 | 0.70 | [0.4, 1.2] | 0.21 |  |
| *Total fats (g)* | -0.07 | 0.06 | 0.93 | [0.84, 1] | 0.22 |  |
| *Linolenic acid (ALA) (g)* | -0.10 | 0.31 | 0.91 | [0.49, 1.7] | 0.75 |  |
| *linoleic acid (LA) (g)* | 0.15 | 0.18 | 1.20 | [0.82, 1.6] | 0.39 |  |
| *Docosahexaenoic acid (DHA) (mg)* | 0.00 | 0.00 | 1.00 | [1, 1] | 0.44 |  |
| *Eicosapentaenoic acid (EPA) (mg)* | -0.00 | 0.00 | 1.00 | [0.99, 1] | 0.33 |  |
| *Saturated fats (SFA) (g)* | 0.00 | 0.09 | 1.00 | [0.84, 1.2] | 0.98 |  |
| *Polyunsaturated fats (PUFA) (g)* | 0.01 | 0.12 | 1.00 | [0.79, 1.3] | 0.97 |  |
| *Monounsaturated fats (MUFA) (g)* | 0.07 | 0.12 | 1.10 | [0.85, 1.3] | 0.54 |  |
| *Cholesterol (mg)* | -0.01 | 0.01 | 0.99 | [0.99, 1] | 0.31 |  |
| *Iodine (µg)* | 0.01 | 0.01 | 1.00 | [0.99, 1] | 0.30 |  |
| *Sodium (mg)* | 0.00 | 0.00 | 1.00 | [1, 1] | 0.62 |  |
| *Calcium (mg)* | -0.00 | 0.00 | 1.00 | [1, 1] | 0.59 |  |
| *Magnesium (mg)* | -0.00 | 0.00 | 1.00 | [0.99, 1] | 1.00 |  |
| *Phosphorus (mg)* | 0.00 | 0.00 | 1.00 | [1, 1] | 0.13 |  |
| *Iron (mg)* | -0.04 | 0.13 | 0.96 | [0.74, 1.2] | 0.75 |  |
| *Selenium (µg)* | 0.00 | 0.02 | 1.00 | [0.97, 1] | 0.82 |  |
| *Vitamin B2 (mg)* | -0.81 | 1.30 | 0.45 | [0.03, 6.1] | 0.54 |  |
| *Vitamin B12 (µg)* | -0.12 | 0.26 | 0.88 | [0.53, 1.5] | 0.63 |  |
| *Vitamin B9 (µg)* | -0.00 | 0.00 | 1.00 | [0.99, 1] | 0.65 |  |
| *Vitamin B3 (mg)* | 0.09 | 0.08 | 1.10 | [0.92, 1.3] | 0.31 |  |
| *Vitamin C (mg)* | -0.00 | 0.00 | 1.00 | [0.99, 1] | 0.88 |  |
| *Vitamin A (µg)* | 0.00 | 0.00 | 1.00 | [1, 1] | 0.43 |  |
| *Vitamin D (µg)* | 0.07 | 0.12 | 1.10 | [0.85, 1.4] | 0.53 |  |
| *Vitamin E (mg)* | 0.09 | 0.15 | 1.10 | [0.81, 1.5] | 0.56 |  |
| *Flavonoids (mg)* | -0.00 | 0.00 | 1.00 | [1, 1] | 0.24 |  |
| *Isoflavones (mg)* | 0.00 | 0.00 | 1.00 | [1, 1] | 0.09 |  |
| *Antacionines (mg)* | -0.00 | 0.00 | 1.00 | [0.99, 1] | 0.59 |  |
| *Resveratrol (mg)* | -0.20 | 0.89 | 0.82 | [0.14, 4.7] | 0.82 |  |
| *Total polyphenols (mg)* | 0.00 | 0.00 | 1.00 | [1, 1] | 0.35 |  |
| *Fresh fruit(g)* | 0.36 | 0.93 | 1.40 | [0.23, 8.9] | 0.70 |  |
| *Sugar Beverage (g)* | 0.01 | 0.04 | 1.00 | [0.93, 1.1] | 0.78 |  |
| *Legumes(g)* | -0.03 | 0.05 | 0.97 | [0.88, 1.1] | 0.54 |  |
| *Nuts and oil olive(g)* | -0.04 | 0.07 | 0.96 | [0.84, 1.1] | 0.51 |  |
| *Vegetables(g)* | -0.00 | 0.03 | 1.00 | [0.94, 1.1] | 0.92 |  |
| *Eggs (g)* | 0.31 | 0.19 | 1.40 | [0.94, 2] | 0.10 |  |
| *Cheese (g)* | 0.01 | 0.05 | 1.00 | [0.92, 1.1] | 0.81 |  |
| *Pastries and sugars(g)* | 0.01 | 0.03 | 1.00 | [0.96, 1.1] | 0.79 |  |
| *Yogurt and milk(g)* | -0.05 | 0.07 | 0.95 | [0.83, 1.1] | 0.48 |  |
| *Potatoes(g)* | -0.01 | 0.03 | 0.99 | [0.93, 1] | 0.69 |  |
| *Processed food(g)* | 0.01 | 0.03 | 1.00 | [0.95, 1.1] | 0.66 |  |
| *Whole grain(g)* | 0.00 | 0.03 | 1.00 | [0.95, 1.1] | 0.96 |  |
| *Refined cereals(g)* | -0.01 | 0.02 | 0.99 | [0.95, 1] | 0.68 |  |
| *Fish(g)* | 0.09 | 0.08 | 1.10 | [0.94, 1.3] | 0.27 |  |
| *Red meat(g)* | -0.07 | 0.19 | 0.93 | [0.64, 1.3] | 0.71 |  |
| *Whitemeat(g)* | 0.00 | 0.03 | 1.00 | [0.94, 1.1] | 0.89 |  |
| *Processed meat(g)* | -0.01 | 0.05 | 0.99 | [0.9, 1.1] | 0.88 |  |
| *Plant-based diet* | -0.44 | 0.37 | 0.64 | [0.31, 1.3] | 0.24 |  |
| *Western diet* | 0.68 | 0.46 | 2.00 | [0.8, 4.9] | 0.14 |  |
| *Meat diet* | -0.21 | 0.40 | 0.81 | [0.37, 1.8] | 0.61 |  |
| *SHEI* | 0.02 | 0.05 | 1.00 | [0.92, 1.1] | 0.71 |  |
| *SHEIQ* | -0.19 | 0.84 | 0.83 | [0.16, 4.3] | 0.83 |  |
| *MDQ* | -0.10 | 0.73 | 0.90 | [0.22, 3.8] | 0.89 |  |
| *DII* | 0.54 | 0.44 | 1.70 | [0.73, 4.1] | 0.22 |  |
| *DIIQ* | 0.10 | 0.42 | 1.10 | [0.49, 2.5] | 0.80 |  |
| *Age* | -0.17 | 0.60 | 0.84 | [0.26, 2.7] | 0.78 |  |
| *Sex* | -0.40 | 0.59 | 0.67 | [0.21, 2.1] | 0.50 |  |
| *Antibiotics* | -1.30 | 1.30 | 0.27 | [0.02, 3.5] | 0.32 |  |
| *z-BMI* | 0.38 | 0.52 | 1.50 | [0.53, 4] | 0.47 |  |

Abbreviations: Exp (β) = Exponential of the coefficient β, representing an odds ratio; CI: Confidence intervals

| **Table S15.** Variable summary in Elastic Net comparison for selected taxa and dietary indexes in the adolescent group | | | | | |
| --- | --- | --- | --- | --- | --- |
| Variable | β-coefficients | Standard Error | **Exp(β)** | CI (95%) | *P value* |
| *Butyricimonas virosa* | -0.45 | 0.21 | 0.64 | [0.42, 0.97] | 0.04 |
| *Parafannyhessea umbonata* | -0.08 | 0.23 | 0.92 | [0.58, 1.5] | 0.72 |
| *Parolsenella catena* | -0.74 | 0.26 | 0.48 | [0.28, 0.8] | 0.00 |
| *Ruminococcus champanellensis* | 0.59 | 0.22 | 1.80 | [1.2, 2.8] | 0.01 |
| *Anaerostipes rhamnosivorans* | 0.48 | 0.22 | 1.60 | [1, 2.5] | 0.03 |
| *Age* | -0.37 | 0.36 | 0.69 | [0.34, 1.4] | 0.30 |
| *Sex* | -0.29 | 0.42 | 0.75 | [0.33, 1.7] | 0.49 |
| *z-BMI* | 0.51 | 0.34 | 1.70 | [0.86, 3.3] | 0.13 |
| *Total fiber (g)* | -0.54 | 1.00 | 0.58 | [0.08, 4.2] | 0.59 |
| *Insoluble fiber (g)* | 0.20 | 1.00 | 1.20 | [0.17, 8.9] | 0.84 |
| *DHA (mg)* | 0.33 | 0.21 | 1.40 | [0.91, 2.1] | 0.13 |

Abbreviations: Exp (β) = Exponential of the coefficient β, representing an odds ratio; CI: Confidence intervals

| **Table S16.** Variable Summary in Elastic Net comparison for selected taxa and food groups in the adolescents group | | | | | |
| --- | --- | --- | --- | --- | --- |
| Variable | β-coefficients | Standard Error | **Exp(β)** | CI (95%) | *P value* |
| *Butyricimonas virosa* | -0.53 | 0.21 | 0.59 | [0.39, 0.9] | 0.01 |
| *Parafannyhessea umbonata* | -0.04 | 0.24 | 0.96 | [0.6, 1.5] | 0.86 |
| *Parolsenella catena* | -0.76 | 0.26 | 0.47 | [0.28, 0.79] | 0.00 |
| *Ruminococcus champanellensis* | 0.58 | 0.22 | 1.80 | [1.2, 2.8] | 0.01 |
| *Anaerostipes rhamnosivorans* | 0.46 | 0.22 | 1.60 | [1, 2.4] | 0.04 |
| *Age* | -0.43 | 0.35 | 0.65 | [0.33, 1.3] | 0.22 |
| *Sex* | -0.18 | 0.42 | 0.83 | [0.37, 1.9] | 0.67 |
| *Antibiotics* | -0.43 | 0.81 | 0.65 | [0.13, 3.2] | 0.60 |
| *z-BMI* | 0.44 | 0.35 | 1.60 | [0.78, 3.1] | 0.21 |
| *Legum* | -0.51 | 0.22 | 0.60 | [0.39, 0.92] | 0.02 |
| *Potatoes* | 0.22 | 0.20 | 1.20 | [0.84, 1.8] | 0.28 |
| *Fish* | 0.39 | 0.22 | 1.50 | [0.96, 2.3] | 0.07 |

Abbreviations: Exp (β) = Exponential of the coefficient β, representing an odds ratio; CI: Confidence intervals

| **Table S17.** Variable summary in Elastic Net comparison for selected taxa and dietary indexes in the adolescent group | | | | | |
| --- | --- | --- | --- | --- | --- |
| Variable | β-coefficients | Standard Error | **Exp(β)** | CI (95%) | *P value* |
| *Butyricimonas virosa* | -0.52 | 0.22 | 0.60 | [0.39, 0.92] | 0.02 |
| *Parafannyhessea umbonata* | -0.21 | 0.23 | 0.81 | [0.52, 1.3] | 0.36 |
| *Parolsenella catena* | -0.59 | 0.25 | 0.55 | [0.34, 0.91] | 0.02 |
| *Ruminococcus champanellensis* | 0.65 | 0.22 | 1.90 | [1.2, 3] | 0.00 |
| *Anaerostipes rhamnosivorans* | 0.53 | 0.22 | 1.70 | [1.1, 2.6] | 0.02 |
| *Age* | -0.39 | 0.38 | 0.68 | [0.32, 1.4] | 0.30 |
| *Sex* | -0.32 | 0.42 | 0.72 | [0.32, 1.7] | 0.45 |
| *Antibiotics* | -0.59 | 0.83 | 0.56 | [0.11, 2.8] | 0.48 |
| *z-BMI* | 0.57 | 0.35 | 1.80 | [0.89, 3.5] | 0.10 |
| *SHEI* | 0.01 | 0.04 | 1.00 | [0.94, 1.1] | 0.84 |
| *SHEIQ* | 0.05 | 0.60 | 1.00 | [0.32, 3.4] | 0.94 |
| *MD* | 0.08 | 0.15 | 1.10 | [0.81, 1.5] | 0.58 |
| *MDQ* | -0.29 | 0.66 | 0.75 | [0.2, 2.7] | 0.66 |
| *DII* | 0.21 | 0.14 | 1.20 | [0.94, 1.6] | 0.13 |
| *DIIQ* | 0.04 | 0.29 | 1.00 | [0.59, 1.8] | 0.90 |

Abbreviations: Exp (β) = Exponential of the coefficient β, representing an odds ratio; CI: Confidence intervals

| **Supplementary S18.** Variable summary in Elastic Net comparison for selected taxa and dietary patterns in the adolescent group | | | | | |
| --- | --- | --- | --- | --- | --- |
| Variable | β-coefficients | Standard Error | **Exp(β)** | CI (95%) | *P value* |
| *Butyricimonas virosa* | -0.49 | 0.21 | 0.61 | [0.41, 0.93] | 0.02 |
| *Parafannyhessea umbonata* | -0.15 | 0.23 | 0.86 | [0.55, 1.3] | 0.51 |
| *Parolsenella catena* | -0.52 | 0.25 | 0.60 | [0.37, 0.97] | 0.04 |
| *Ruminococcus champanellensis* | 0.64 | 0.22 | 1.90 | [1.2, 2.9] | 0.00 |
| *Anaerostipes rhamnosivorans* | 0.48 | 0.22 | 1.60 | [1.1, 2.5] | 0.03 |
| *Age* | -0.38 | 0.35 | 0.69 | [0.34, 1.4] | 0.28 |
| *Sex* | -0.14 | 0.43 | 0.87 | [0.38, 2] | 0.75 |
| *Antibiotics* | -0.64 | 0.82 | 0.52 | [0.11, 2.6] | 0.43 |
| *z-BMI* | 0.59 | 0.35 | 1.80 | [0.9, 3.6] | 0.09 |
| *Plant-based diet* | -0.39 | 0.26 | 0.68 | [0.41, 1.1] | 0.14 |
| *Western diet* | 0.56 | 0.28 | 1.80 | [1, 3] | 0.04 |

Abbreviations: Exp (β) = Exponential of the coefficient β, representing an odds ratio; CI: Confidence intervals
